# Pattern formation along signaling gradients driven by active droplet behavior of cell swarms

**DOI:** 10.1073/pnas.2419152122

**Published:** 2025-05-20

**Authors:** Hugh Z. Ford, Giulia L. Celora, Elizabeth R. Westbrook, Mohit P. Dalwadi, Benjamin J. Walker, Hella Baumann, Cornelis J. Weijer, Philip Pearce, Jonathan R. Chubb

**Affiliations:** ^a^Institute for the Physics of Living Systems, University College London, United Kingdom; ^b^Laboratory for Molecular Cell Biology, University College London, London WC1E 6BT, United Kingdom; ^c^Department of Mathematics, University College London, London WC1H 0AY, United Kingdom; ^d^Department of Mathematical Sciences, University of Bath, Bath BA2 7AY, United Kingdom; ^e^Intelligent Imaging Innovations Ltd, 17 Westbourne Studios, London W10 5JJ, United Kingdom; ^f^Division of Molecular, Cell and Developmental Biology, School of Life Sciences, University of Dundee, Dundee DD1 5EH, United Kingdom

**Keywords:** signaling gradients, active droplet, chemotaxis, tissue fluidity, pattern formation

## Abstract

Gradients of extracellular signals organize cells in tissues. Although there are several models for how gradients can pattern cell behavior, it is not clear how cells react to gradients when the population is undergoing 3D morphogenesis, in which cell–cell and cell–signal interactions are continually changing. *Dictyostelium* cells follow gradients of their nutritional source to feed and maintain their undifferentiated state. Using lightsheet imaging to simultaneously monitor signaling, single-cell, and population dynamics, we show that the cells migrate toward nutritional gradients in swarms. As swarms advance, they deposit clumps of cells at the rear, triggering differentiation. Clump deposition is explained by a physical model in which cell swarms behave as active droplets: cells proliferate within the swarm, with clump shedding occurring at a critical population size, at which cells at the rear no longer perceive the gradient and are not retained by the emergent surface tension of the swarm. The model predicts vortex motion of the cells within the swarm emerging from the local transfer of propulsion forces, a prediction validated by 3D tracking of single cells. This active fluid behavior reveals a developmental mechanism we term “musical chairs” decision-making, in which the decision to proliferate or differentiate is determined by the position of a cell within the group as it bifurcates.

## Significance

Gradients of extracellular signals are used to organise the distribution of different cell types within tissues. It is unclear how cells adopt different cell types when they are migrating along gradients. We use lightsheet imaging of Dictyostelium cells to follow cell type choice together with the dynamics of their nutritional signaling gradient. Our data show that cells follow the gradient as a swarm, with cells entering the differentiation programme by being shed, as dense clumps, by the swarm. The shedding of groups and flow patterns of cells within the swarm imply cells behave as a living active droplet, with these fluid-derived behaviours determining the overall spatial organisation of differentiated and undifferentiated cell states in this migratory population.

Signaling gradients are interpreted by cells to guide their migration and to direct the subdivision of embryonic tissues into specific cell types (1, 2). Despite the widespread functioning of gradients in both development and disease, it has remained challenging to monitor natural signaling gradients together with cell and tissue responses over time. In contexts with limited tissue reorganization, it has been possible to infer how cells react to signal gradients (3). However, for contexts in which three-dimensional tissue organization remodels substantially over time, there are significant barriers to interpreting the connection between signal inputs and behavioral outputs of cells. In these systems, the organization of cells continually changes, influencing and being influenced by cell–cell interactions (4, 5) and extracellular signal gradients (6–11) in addition to any emergent tissue properties, which all combine to influence the cell response to signaling.

In this study, we investigate the emergent dynamics and organization of cell groups migrating toward self-generated signaling gradients. We use light sheet imaging to simultaneously monitor the dynamics of a nutritional signaling gradient and its effects on the migration and differentiation of populations of *Dictyostelium* cells. We show how the gradient organizes single cells into dense groups—swarms. These swarms periodically shed large cell clumps, driving the cells in the clumps into the developmental program. Clump shedding is surprising in the light of traditional models of collective cell chemotaxis along self-generated gradients, which predict continuous, rather than periodic, cell shedding (12, 13). To explain this emergent behavior, we developed and tested a coarse-grained mathematical model in which the cell swarm is represented as an active droplet. Our model implies that emergent material properties are a key determinant of pattern formation in these chemotactic cell populations. The model also predicts an emergent vortex motion of cells within the swarm, which our experiments confirm is a key driver of cell transport. Behaviors of the swarm arising from droplet properties (shedding and vortex motion) combine to determine cell fate: the position of the cell in the vortex at the time of clump shedding dictates whether or not the cell enters the developmental program.

## Results

### Shedding from Cell Swarms During Chemotaxis.

**Fig. 1.**
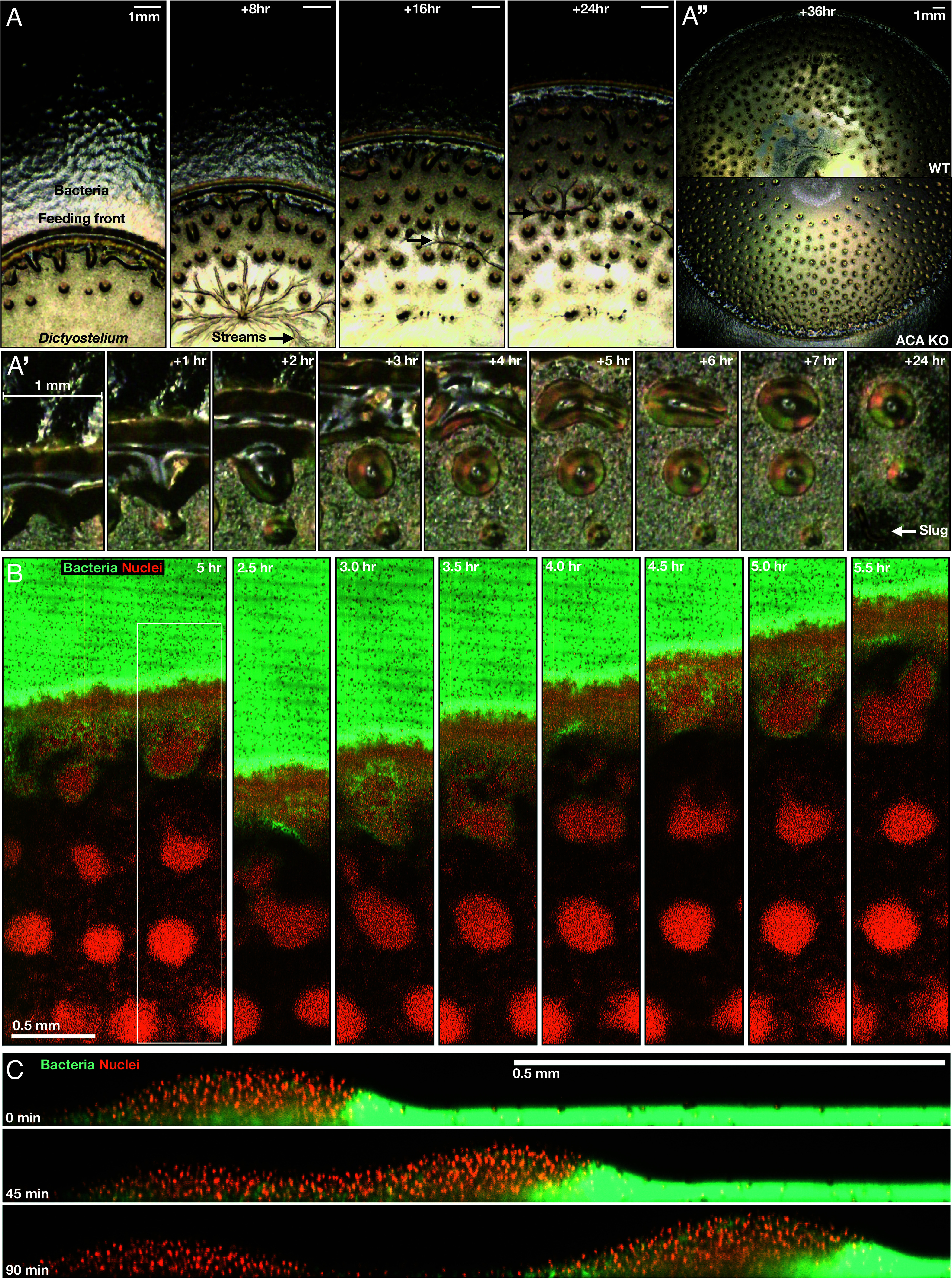
Multiscale imaging of cell migration to signaling gradients. (*A*) Macrophotography of the feeding front, see also Movie S1. *Dictyostelium* cells progressively clear the bacteria as an expanding ring-shaped band. Cells are left behind (inside the ring) as isolated cells and compact cell clumps. Arrows show later aggregation events. (*A*′) Close-ups of 2 cell shedding events. (*A*″) Clump shedding is unperturbed in *acaA*- cells (no cAMP signaling), and clumps are more persistent. Images show a comparison of feeding fronts of wild-type and *acaA*- cells. (*B*) 3D imaging (light sheet) of feeding fronts. Images of fluorescently labeled bacteria (green) and *Dictyostelium* cell nuclei (red) at the feeding front, see also Movie S3. The right and central panels show the progression of the feeding front and clump formation within the region marked by the white box in the *Left* panel. (*C*) Similar to *B*, but showing a side view of the feeding front at 45-min intervals, see also Movie S4.

*Dictyostelium* cells use signaling gradients to coordinate their differentiation program. In their undifferentiated proliferative state, these soil-dwelling amoebae locate their nutritional source, bacteria, by chemotaxis toward bacterial metabolites (14). Without bacteria, the cells starve and enter their developmental program, in which single cells form multicellular aggregates via chemotaxis toward cAMP, before forming a final structure carrying dormant spores. To mimic natural environments, we spotted cells on lawns of their bacterial food source (15–17). Macrophotography shows the proliferating cell population clearing the bacterial lawn as an advancing ring-shaped band, called the feeding front (Fig. 1*A* and Movie S1). Compact clumps are shed from the feeding front, which collectively form a spotted pattern (*SI Appendix*, Fig. S1*A*). These clumps emerge from patches along the advancing feeding front that elongate then pinch off and round up into isolated domes (Fig. 1*A′*, *SI Appendix*, Fig. S1*C*, and Movie S2). Further from the feeding front are the first clear signs of development: cell streaming and aggregation, characteristic of cAMP chemotaxis (Fig. 1 *A*, second panel and Fig. 1 *A*′, last panel).

To investigate the cell–cell and cell–signal interactions at the feeding front requires distinguishing between *Dictyostelium* cells and the bacteria. For this, we used light sheet microscopy to live image fluorescently labeled bacteria and cell nuclei at the millimeter scale over multiple hours (Fig. 1 *B* and *C*, *SI Appendix*, Fig. S2, and Movies S3 and S4). These data show that the cells advance at the interface with the bacteria as a densely packed and highly motile group—a “swarm.” This behavior is characteristic of classic Keller–Segel models of chemotaxis (12, 13), in which cell groups locally degrade a chemoattractant, resulting in a “self-generated” chemoattractant gradient. The cell groups migrate along the gradient, continually shifting the gradient by degradation as they move, while remaining at a constant size due to a balance of cell growth and continuous cell shedding (18, 19). However, as implied by the macrophotography (Fig. 1*A*), and in contrast to predictions from classic Keller–Segel models, in addition to continuous cell shedding (Movies S3 and S4), most cells (~60 to 70%) are left behind in large, stable, and spatially compact clumps that are distinct from the field of isolated cells (Fig. 1 *B* and *C* and *SI Appendix*, Fig. S3). The cell clumps do not reengage with the advancing front, indicating that the cells within them are destined to starve and then enter the developmental program.

Multicellular development in *Dictyostelium* is dependent on the chemoattractant cAMP. To test whether the shedding of cell clumps requires cAMP, we analyzed clump shedding in cells lacking *acaA*, the gene encoding the adenylyl cyclase synthesizing cAMP during starvation (Fig. 1*A*″, *SI Appendix*, Fig. S1*B*, and Movie S1). In *acaA-* cells, clump shedding occurs with the same characteristics as wild-type, indicating shedding does not require cAMP. Indeed, without cAMP, clumps are abnormally persistent, implying cAMP is required for clump dispersal, not formation. The breaking up of clumps is necessary for the transition to the multicellular structures of later stages of the wild-type developmental program (Fig. 1*A*′, *SI Appendix*, Fig. S1*C*, and Movies S1 and S2). Wild-type clumps withstand multiple rounds of cAMP signaling by neighboring cells before they disperse, evidenced by these neighbors undergoing extensive streaming while recently shed clumps remain intact. Indeed, the mean clump lifetime is around 24 h, compared to the onset of cAMP signaling at around 4 to 6 h for dispersed cells (20). Consequently, development is suspended by around a day for the cells in clumps compared to cells outside clumps. This spontaneous heterogeneity in developmental timing may provide flexibility within the population to counter uncertain nutrient availability or variance in the opportunity to disperse spores.

### Clump Shedding Follows Gradient Dynamics.

**Fig. 2.**
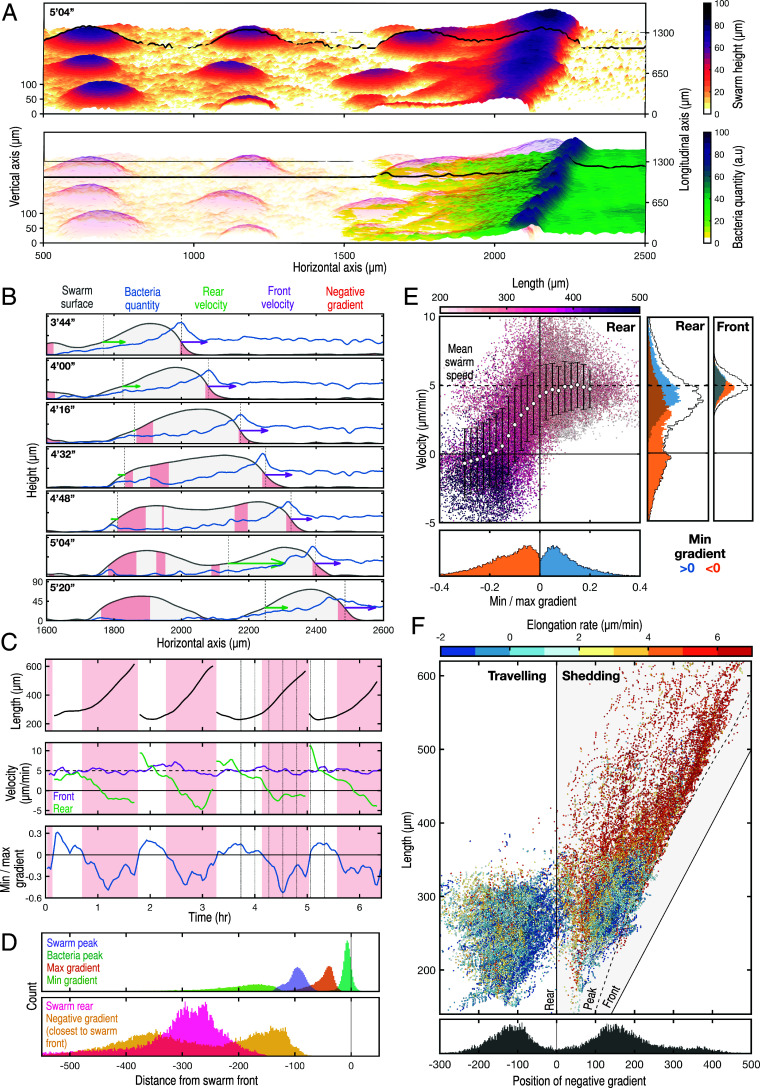
Coupled swarm and signaling gradient dynamics trigger collective cell shedding. (*A*) Quantification of the swarm height and bacteria quantity at the feeding front (from data in Fig. 1*B*). Black lines mark the swarm boundary (*Top* panel) and bacterial quantity (*Bottom* panel) at one cross section. (*B*) Swarm shape dynamics during clump shedding. Time-lapse (h′min″) of cross sections of the swarm height (gray) and bacteria quantity (blue) from *A* (black line) throughout a shedding event. Also shown: the regions where the gradient is negative (pink), swarm front and rear (black dotted lines, where swarm height = 30 µm), and velocity vectors of the swarm front (purple) and rear (green). (*C*) Tracking the dynamics of swarm length, velocity, and bacterial gradient for a cross-section of the feeding front, through several shedding cycles. Shedding events are defined as a collapse in swarm length. Swarm elongation is summarized by differences in velocity of the swarm front (purple) and rear (green). The bacterial gradient is summarized as the ratio of the minimum gradient and maximum gradient within the swarm. Times where the minimum gradient is negative shown as pink blocks. Vertical lines in the third shedding cycle correspond to sequence of plots in *B*. The mean swarm speed (4.9 µm/min) is shown as a black dashed line. (*D*) The bacterial peak always coincides with the swarm front. Plots summarize gradient and swarm properties at all time and spatial points. Top plot shows distances between the locations of swarm front (black line), the swarm and bacterial peaks, and the maximum and minimum gradient within the swarm. The bottom plot shows how the zero gradient can be positioned either side of the swarm rear. (*E*) Elongation (reduction of rear velocity) only occurs when the minimum gradient becomes negative within the swarm. The main plot shows the relationship between the rear velocity, length (color), and gradient (ratio of the minimum and maximum gradient). Error bars show SD of rear velocity at different gradients. The *Bottom* panel is a frequency distribution showing the gradient partitions into positive and negative values. *Side* panels (frequency distributions) show the front speed is independent of the minimum gradient, whereas the rear speed changes from the mean swarm speed to around zero when the minimum gradient becomes negative. *Bottom* and *Side* panels share axes with the main panel. (*F*) Two phases of swarm behavior: traveling and shedding. The *Top* panel shows two clusters of swarm behavior differing in the position of the negative gradient relative to the swarm rear, swarm length, and elongation rate (color) for all spatial and time points. The position of the negative gradient is defined as the closest negative value to the swarm peak. *Bottom* plot: two discrete clusters showing locations of the negative gradient either behind (left cluster—negative) or deep within the swarm boundary (right cluster—positive).

Based on this initial analysis, we infer that the shedding of clumps from the feeding front emerges from physical interactions between cells and/or interactions between cells and the bacterial gradient. To determine how swarm motion and clump shedding relate to the gradient, we quantified the dynamics of swarm size together with the distribution of bacteria (Fig. 2*A*, *SI Appendix*, Figs. S2*B* and S3*A*, and Movies S3 and S5). Based on standard models of a migrating population sensing a self-generated gradient, one might expect a stable exponential or logistic decay in the quantity of bacteria from high at the front to low at the rear of the swarm (10, 12, 18, 19); we define this as a positive gradient. However, our data show that the bacterial gradient is highly dynamic and can flatten and even reverse toward the rear of the swarm—in other words, a minimum gradient ≤ 0 within the swarm boundary (Fig. 2 *B* and *C*—pink shading, *SI Appendix*, Figs. S4 and S5*B*). Additionally, as observed previously (15), bacteria accumulate along the feeding front, creating a local peak with up to twice the quantity of bacteria found ahead of the swarm (Fig. 2*B* and *SI Appendix*, Fig. S5*A*). While cell feeding on a bacterial lawn can explain the flattening of bacteria gradients at the rear, it can not account for the local peak in bacteria ahead of the swarm. To understand the basis of the bacteria peak, we used particle image velocimetry (PIV) to quantify the bulk motion and interactions of the swarm and bacteria (*SI Appendix*, Fig. S6 *A* and *B* and Movie S4). This analysis implies the bacteria are pushed forward, with the swarm acting analogous to a snowplow, suggesting the bacterial population possesses a material integrity that provides resistance to swarm penetration (*SI Appendix*, Fig. S6*C*). The bacteria peak remains a constant size at the swarm front, consistent with a balance between accumulation via swarm motion and degradation via feeding. This persistent bacteria accumulation creates a robust, positive bacteria gradient localized at the swarm front (Fig. 2 *B* and *D*), potentially directing long-range migration for cells toward the leading edge. The gradient is not necessarily positive toward the rear, with zero/negative gradients occurring at varying positions with respect to the rear of the swarm (Fig. 2 *B* and *D* and *SI Appendix*, Figs. S4*B* and S5*B*). Overall, these results imply the swarm shapes the chemoattractant gradient by both spatially reorganizing and degrading the bacteria.

Tracking the dynamics of swarm size and bacterial gradient for a cross-section of the feeding front, through several shedding cycles (Fig. 2*C* and *SI Appendix*, Fig. S4), reveals how clump shedding is preceded by i) steady swarm elongation, ii) a reduction of the rear velocity of the swarm to around zero and iii) the emergence of a local negative bacterial gradient within the swarm boundary. Indeed, comparing these three quantities across whole datasets shows that the swarm rear becomes stationary only once the minimum gradient within the swarm is negative (Fig. 2*E*). Incorporating spatial information (tracking the position of the furthest forward negative gradient) into this analysis reveals two clusters corresponding to two phases of swarm dynamics: traveling and shedding (Fig. 2*F*). On the plot, the position of the negative gradient zone relative to the swarm rear is defined such that the position of the zone is positive when it is within the swarm, and negative when behind. The traveling phase is characterized by a compact swarm (length below 350 µm) with a positive minimum gradient within the swarm (Fig. 2*F*). In this phase, the swarm size remains stable because cells at the rear move at a similar speed to cells at the front, causing no swarm elongation (Fig. 2*E* and *SI Appendix*, Fig. S5*C*). This is consistent with all cells in the swarm having access to a positive bacterial gradient and therefore adequate positional information on the location of the bacterial food source. In contrast, the shedding phase is characterized by an increased swarm elongation rate and a negative gradient deep within the swarm boundary (Fig. 2 *E* and *F* and *SI Appendix*, Fig. S5*D*). This rapid elongation is consistent with cells at the swarm rear lacking positional information derived from the bacterial front—the swarm elongates because the cells at the back reduce their motility while those at the front keep moving.

What causes the loss of positional information within the swarm and why does this cause collective shedding? As might be expected due to cell growth and proliferation, our data show a steady and low baseline rate of swarm elongation during the traveling phase (*SI Appendix*, Fig. S5*C*). Swarms above a critical length (350 µm) then rapidly elongate and eventually split (Fig. 2*F* and *SI Appendix*, Fig. S5 *C* and *E*), suggesting cells at the swarm rear lose positional information because they are too far from the bacterial source. However, this interpretation does not account for the bulk shedding of cell clumps, because continuous cell growth would steadily push cells beyond the critical length at the rear, resulting in a continuous shedding of cells, as predicted by classic Keller–Segel models of chemotaxis (12, 13). Alternatively, the redistribution of bacteria across the swarm (*SI Appendix*, Fig. S6*C* and Movie S4) could cause the sudden emergence of a negative gradient, with the associated loss of positional information from the front. However, swarms initially maintain compactness beyond the appearance of a negative gradient at the swarm rear (Fig. 2*F*—bottom of shedding cluster). Indeed, even during splitting events, the swarm maintains a smooth and consistent boundary until it pinches off (Fig. 2*B* and *SI Appendix*, Figs. S4*B* and S5*D*). This suggests that some emergent material property of the multicellular swarm combines with growth and loss of positional information to trigger the transition to shedding.

### Active Fluid Model of Cell Swarms Captures Periodic Shedding.

**Fig. 3.**
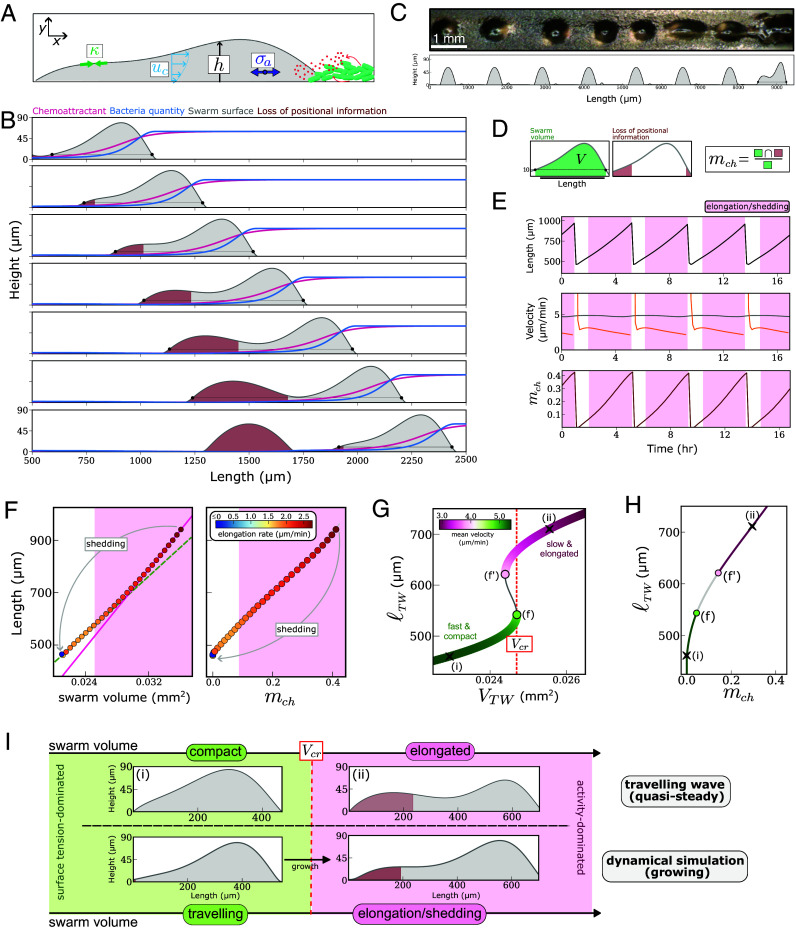
Active fluid model of cell swarms. (*A*) Schematic summarizing the key features of the active fluid model (Modeling Supplement). (*B*) Time-lapse (25-min intervals) of the simulation of the active fluid thin-film model. We plot the swarm height (gray curve), bacteria quantity (blue curve) and the normalized concentration of chemoattractant (red curve). In brown is the region where the gradient in the chemoattractant drops below a cell sensitivity threshold (*SI Appendix*, *Mathematical Modeling*). The black dots show the points at which the swarm height drops below a 10 μm threshold. (*C*) Comparison between 1D-track experiment (*Top* figure; viewed from above) and model simulations of a swarm cross section (*Bottom* figure). The model captures the periodic shedding of clumps and quantitatively matches their distance (1.25 mm) and shedding rate (4.6 h). (*D*) Schematic summarizing how we extract swarm length and swarm volume from the output of the dynamic simulation of the active fluid model. The metric, $m_{\mathrm{ch}}$, is obtained as the ratio between the swarm volume that lacks positional information, which is given by the intersection between the green and red area in the figure, and the total swarm volume. (*E*) The length (black), velocity of the swarm front (gray) and rear (orange), and the ratio of the swarm mass exposed to a chemoattractant gradient below the cell sensitivity threshold ($m_{\mathrm{ch}}$, dark red), throughout four shedding cycles. (*F*) The two phases (traveling and elongation/shedding) of swarm dynamics shown via the relationship between swarm length and normalized swarm mass (*Left* plot) and the ratio of the swarm mass exposed to a chemoattractant gradient below the cell sensitivity threshold $m_{\mathrm{ch}}$ (*Right* plot). During the traveling phase, the length-to-mass ratio slowly increases (green dashed line in the *Left* panel). Transition to swarm elongation is signified by an increase in the length-to-mass ratio (magenta line in the left plot) and the key swarm quantity $m_{\mathrm{ch}}$ increasing above a critical (nonzero) threshold. In both plots, the pink shaded area indicates the elongation/shedding phase. (*G*) Bifurcation diagram of traveling wave (TW) solution for the active fluid droplet model illustrating how the length of the swarm (colored by the swarm TW velocity) changes as a function of the swarm mass. Bifurcation points (f and f′) delimit a region of bistability. The presence of the twofold points introduces a discontinuous transition in the mode of swarm migration which is controlled by the volume increasing above the critical value, $V_{\mathrm{cr}}.$ We expect the swarm to transition from a “fast and compact” phenotype to a “slow and elongated” phenotype as the swarm size increases above $V_{\mathrm{cr}}$ (see panel *I*). (*H*) Two types of TW swarm solution shown via the relationship between the length of the swarm and the metric $m_{\mathrm{ch}}$. (*I*) Schematic illustrating the connection between the traveling wave analysis and the two phase regime observed in the dynamical simulations. Top plots showing the height profile for the traveling wave solutions corresponding to point (i) and (ii) in the bifurcation diagram (*F*). Bottom plots showing the height profile for the swarm front in the dynamical simulations (extracted from first and third panels in *B*).

Based on the high density of cells, the clearly delineated swarm boundary and the rounding of clumps following shedding implying a surface tension (Fig. 1*A* and *SI Appendix*, Fig. S1*A*), we reasoned that the swarm has emergent fluid-like properties caused by physical interactions between cells. That is, we interpret the cell swarm as a living active droplet: a viscous fluid that grows and moves (21–24). To investigate whether and how emergent fluid-like properties determine the observed swarm dynamics, we developed a continuum, coarse-grained, active fluid thin-film model for a cross-section of the swarm (Fig. 3*A* and *SI Appendix*, *Mathematical Modeling*).

In the model, we assumed that the following three material properties contribute to emergent mechanical stress in the swarm: i) an effective surface tension κ, which generates capillary stresses that confine the advancing cell swarm, and give a propensity for circularity and consistent contact angles in stationary cell clumps (*SI Appendix*, Fig. S3 *B*–*D*); ii) an effective viscosity η which generates viscous stresses; this property arises from turnover of cell–cell attachments and rearrangements of cells within the swarm (25); iii) an activity parameter ξ, associated with an effective active contribution $\sigma_{a}$ to the stress in the fluid, which arises from the alignment of directed cell motion due to chemotactic bias. Cell proliferation was modeled through film growth, which is mediated by the local concentration of bacteria. In the model, bacteria are consumed by cells and produce diffusible chemoattractant molecules that decay at a constant rate. To capture the emergent flow of cells inside the swarm, we used lubrication theory, which is valid for long, thin films appropriate for the geometry of the swarms studied here (Figs. 1 and 2). We applied an effective Navier slip condition at the floor to account for effective friction with the floor (26), which also captures the impedance of cell motion by the bacteria lawn revealed by the PIV data (see *SI Appendix*, *Mathematical Modeling* for a more detailed discussion of the appropriate boundary condition). Under these assumptions, the flow of cells in the swarm, $u_{c}$, has a parabolic profile (Fig. 3*A*); the magnitude of the flow depends on the relative sizes of surface tension, viscosity, and active stress gradients. The model suggests that the emergent flow field causes the swarm to migrate up self-generated chemoattractant gradients, which are in turn shaped by feeding (Fig. 3*B*).

To calibrate the model, we estimated the emergent material properties of the swarm (*SI Appendix*, *Mathematical Modeling*), by quantitatively matching model predictions for the shedding rate and distance between shed clumps to experimental data (Fig. 3*C* and *SI Appendix*, *Mathematical Modeling*). For this purpose, we used macrophotography to live image feeding fronts constrained to thin lines of bacteria, which enables unambiguous measurement of the shedding rate (Movie S7). In this context, we find that shedding is periodic with a rate of 1 clump per 4.35 h, a similar timescale to the proliferation rate of cells feeding on bacteria (27). Calibrated model simulations recapitulate the two observed phases of the swarm movement: traveling and shedding (Fig. 3 *B*, *E*, and *F* and Movie S6). We conclude that our minimal model can explain the emergent swarm dynamics observed experimentally.

### Emergent Material Properties and Cell Proliferation Explain Periodic Shedding.

We used the calibrated model to further explore the physical mechanisms controlling the observed periodic shedding. During the traveling phase, the swarm rear and front move at a steady velocity (Fig. 3*E*). In contrast, during the elongation phase, the model and data show a rapid increase in swarm length due to the rear of the droplet moving slower than the front (Figs. 1*C*, 2*E*, and 3*E*). This causes an increase in the ratio between the elongation rate and proliferation-driven swarm expansion (Fig. 3 *F*, *Left* panel), suggesting that the elongation phase is not triggered by a sudden increase in proliferation, but rather by a redistribution of the mass within the swarm. Similar transitions from compact to elongated phenotypes are observed in sliding droplets under sufficiently strong gravity and are connected to the pearling phenomenon—the emission of smaller droplets from the moving front (28). While gravity-driven droplets experience a uniform body force, living droplets in our model experience (and shape) spatially varying forcing due to the dependence of the activity term on the local chemoattractant gradient. To better understand how swarm mass is redistributed, we considered a simplified version of our model, in which the swarm is described as a traveling droplet with quasi-constant volume V. In this simplified framework, we find that compact, fast-traveling swarms can only exist for swarm volumes V below a critical value $V_{cr}$ (*SI Appendix*, *Mathematical Modeling*, Fig. 3 *G* and *I*). For swarm volumes above this critical value, the model predicts only elongated, slow-traveling swarms (Fig. 3*G*). For increasing values of V, the compact swarm solution transitions to the elongated solution through a discontinuous phase transition [mathematically a fold bifurcation (29)] at $V=V_{cr}$. The physical explanation for the presence of traveling and elongation phases in our model is as follows: there is a competition between capillary forces generated by surface tension, which favor swarm compactness, and chemotaxis-driven gradients in active stress, which favor the elongation of the swarm (Fig. 3 *F* and *H*). The transition between phases—from a surface tension-dominated (compact) to an activity-dominated (elongated) regime—coincides with the loss of positional information for a large enough volume at the rear of the swarm, which occurs at a critical overall swarm volume (Fig. 3 *D* and *E* and *SI Appendix*, Fig. S5*E*). In the dynamical simulations, the critical volume is reached because of slow cell proliferation; then, the crossing of the bifurcation forces the swarm to reassemble over a timescale much faster than growth, eventually leading to shedding (Fig. 3*I*).

### Perturbing Swarm Material Properties Alters Shedding Morphologies.

**Fig. 4.**
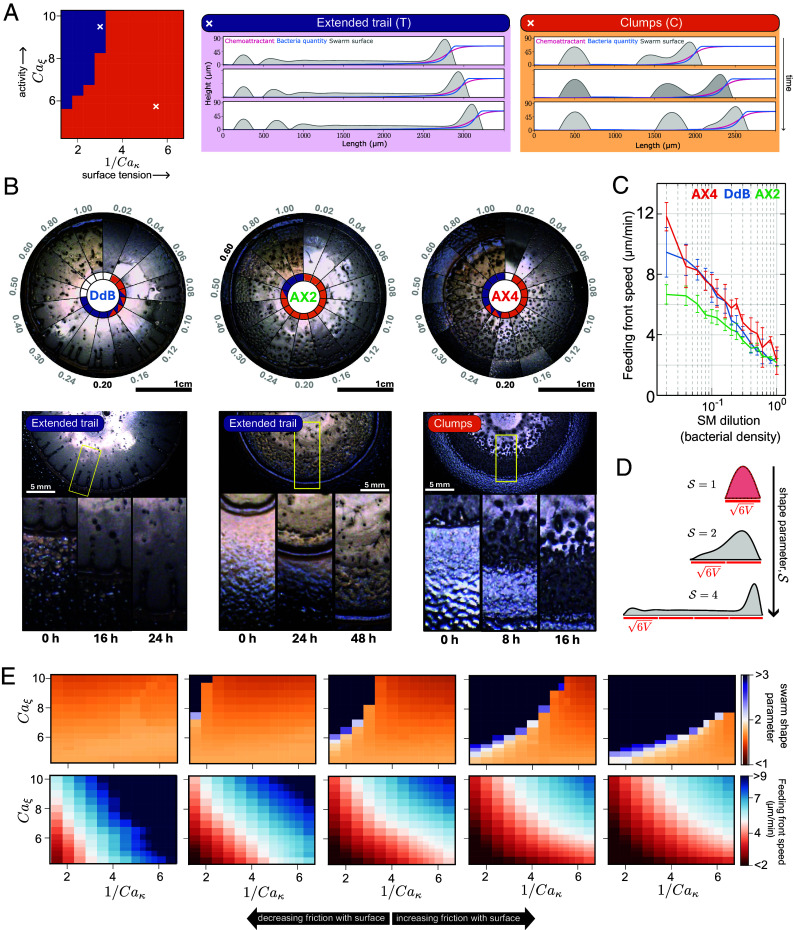
Cell and environmental properties determine different shedding behaviors. (*A*) Model simulations show different shedding behaviors—clumps and extended trails—at different levels of bulk activity and surface tension. *Left*: Phase diagram for the swarm shedding behavior as a function of the passive, ${\mathrm{Ca}}_{\kappa },$ and active, ${\mathrm{Ca}}_{\xi }$, capillary numbers (defined in *SI Appendix*, Eq. **S12a**). *Right*: Characteristic model simulations of the two shedding morphologies: (T) extended trails and (C) clumps; the value of the capillary numbers used for the simulation are indicated with the crosses on the phase diagram. (*B*) Experimental validation of different predicted shedding behaviors. *Top*: Composite images of feeding front behaviors of three different strains: DdB, AX2, and AX4. Each “pie” is divided into segments corresponding to increasing bacterial concentrations (generated by varying the media richness). In the center of each pie, the different shedding behaviors are color-coded to match the colors in *A*. *Bottom*: Close-ups of time-lapses showing different behaviors. DdB shows extended trails with finger-like shapes. AX2 shows extended trails that resemble sheets. AX4 shows clumps. (*C*) Feeding front expansion is slowed at higher bacterial densities for all strains. Feeding front speeds shown for DdB, AX2, and AX4 on different media (SM) strengths. Averages taken from 12 colonies per strain (3 colonies per plate on 4 plates). (*D*) Schematic outlining the definition of the shape parameter S introduced for measuring clump morphology. This is defined as the ratio of the swarm length and $\sqrt{6V}$, which is the length of a passive stationary droplet of equal volume V (*SI Appendix*, *Mathematical Modeling*, section 3.2). The larger the shape parameter at the time of the shedding, the closer the swarm phenotype is to the elongated trail (*A*). (*E*) Model simulations showing that increasing floor friction retards swarm migration and favors transitioning from clump shedding to elongated trails. The panel shows phase diagrams for migrating swarms with variable surface tension and activity at different levels of surface friction. *Top*: Shape parameters for the simulated swarm at the time of shedding; *Bottom*: Average front migration speed for the simulated swarm. From left to right, the surface friction is ×1/4, ×1/2, ×1, ×2, ×4 of the reference value (*SI Appendix*, Table SM2).

In the model, the periodicity of shedding and the critical volume are determined by the emergent material properties of the swarm (Fig. 4*A*, *SI Appendix*, Figs. SM5 and SM7). This dynamical model predicts two different morphologies for the leading front during shedding: shedding via the emission of clumps directly behind the leading front—as in our experiments (Fig. 1)—and shedding from an elongated trail (Fig. 4*A*). To explore whether both these morphologies could be observed in experiments, we tested swarm migration and shedding in a range of experimental conditions, including different *Dictyostelium* isolates and bacterial densities (Fig. 4*B*). In agreement with the model prediction, both morphologies were observed across the experiments, with the elongated trail phenotype manifesting as either fingers or a continuous mat extending from the leading front (the difference between these can not be captured by our pseudo-2D model).

To further explore the mechanisms underlying each shedding behavior, we again iterated between model and experiment. Increasing the bacterial density in the experiments promoted the elongated trail morphology and reduced the overall swarm migration speed (Fig. 4 *B* and *C*). These effects do not appear to be explained by receptor saturation, which, in contrast, decreases the tendency for an elongated trail morphology (*SI Appendix*, Fig. SM8). Informed by the earlier PIV analysis (*SI Appendix*, Fig. S6*C*), we reasoned the observed dynamics could be driven by an increase in impedance to the movement of *Dictyostelium* cells at higher bacterial densities. We tested this hypothesis with our model by varying impedance to motion via changing the values of the effective friction with the floor. To this end, we generated the 3D phase diagram for migrating swarms with variable surface tension, activity, and floor friction (Fig. 4*E*). We find that increasing friction with the floor in the model can indeed capture the slowing down of the feeding front, consistent with the data in Fig. 4*C*. In addition, increasing the friction with the floor in the model also captures the transition from clumps to elongated trail morphology at higher bacterial densities in the experimental data (Fig. 4 *D* and *E*). Overall, these results demonstrate how the emergent material properties of *Dictyostelium* swarms and their physical environment determine migration and pattern formation in response to signaling gradients.

### Implications of Swarm Active Fluid Behavior for Individual Cells.

**Fig. 5.**
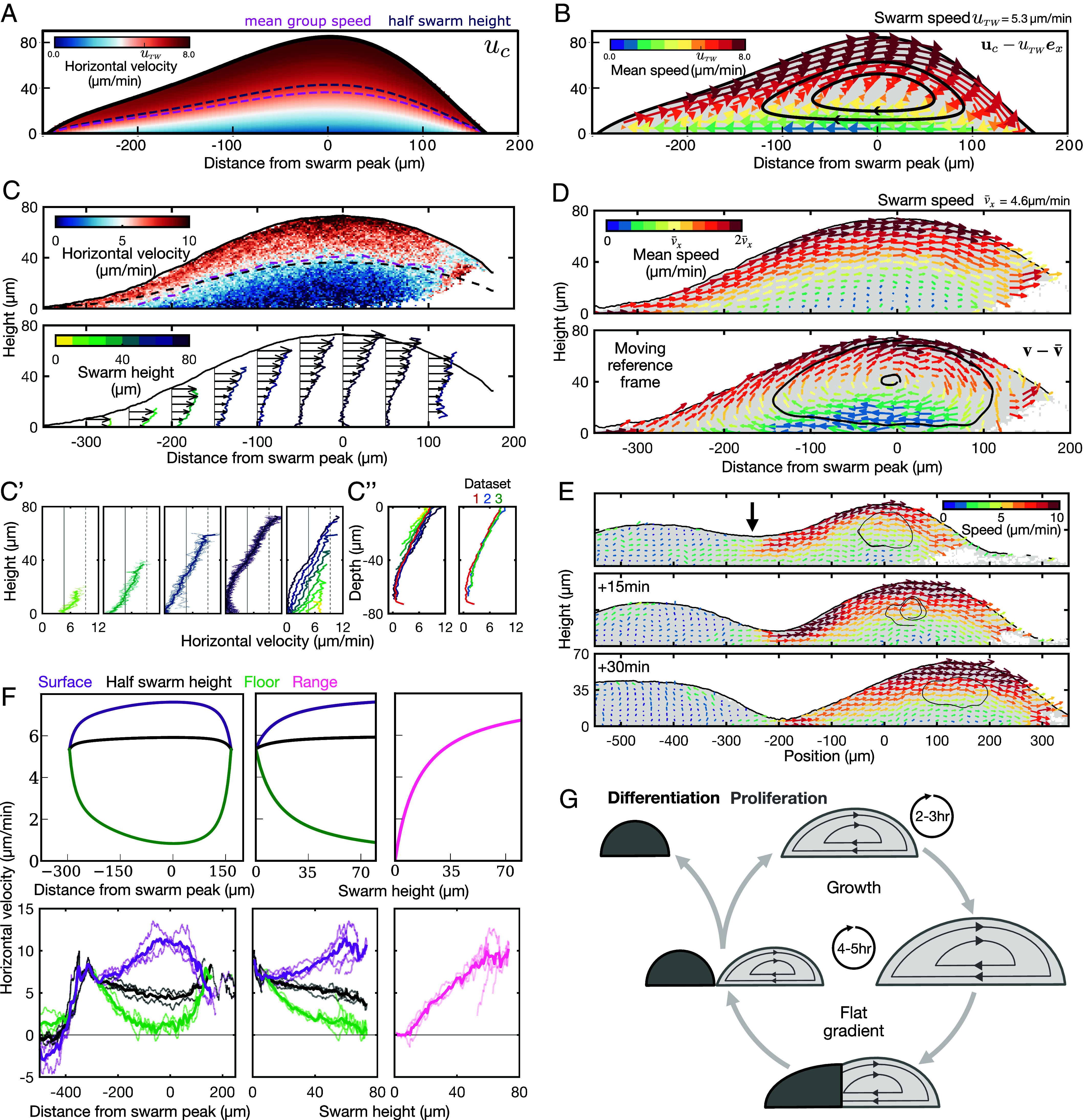
Validating the cell flow patterns predicted by the active fluid model. (*A*) Model predictions for the horizontal velocity field within the swarm, labeled with contours for mean group speed and half swarm height. (*B*) Model predictions for the vector field of cell flows in the moving reference frame of the swarm, labeled with streamlines summarizing cell circulation. The vectors are colored by the magnitude in the stationary reference frame. (*C*) Mean cell horizontal velocity field obtained from cell tracks. Plots also show contours for the half-swarm height (black) and mean group speed (pink). The *Bottom* panel shows vector field of horizontal velocities, summarized with curves colored by swarm height. Panel (*C*′) shows these curves partitioned into different heights, with the *Far Right* panel combining these mean horizontal velocity profiles for different swarm heights. In contrast, in *C*″ these curves are structured by swarm depth rather than height. The *Left* panel is from one dataset; the *Right* panel shows averaged curves from three datasets. (*D*) Validation of model predictions showing vortex flow in the swarm in the stationary (*Top*) and moving (*Bottom*) reference frame. Plots derived from 3D cell tracking data and plotted as in *B*. In both panels, the mean cell velocity vectors are colored by the magnitude in the stationary reference frame. In the *Bottom* panel, two streamlines are plotted to indicate cell circulation. (*E*) Vortex fields derived from 3D cell tracking data during a shedding event. (*F*) Comparing cell velocity profiles between model (*Top* panels) and data (*Bottom* panels). *Left* panels: Mean horizontal cell velocity at the surface, floor, and half swarm height at different distances to the swarm peak. *Middle* panels: Horizontal velocities as a function of swarm height. *Right* panels show the range (distance between surface and floor velocities). (*G*) Musical chairs decision-making. Schematic of the properties of an active droplet that emerge during cell swarming, and influence cell differentiation and proliferation. Within the migrating swarm (light), cells proliferate and circulate with a maximum period of around 2 to 3 h. Once a critical fraction of the swarm experiences a flat gradient (dark), the cells at the swarm rear will collectively cease to migrate and deposit within a cell clump, which are destined for differentiation.

A further key prediction of the active droplet model of migrating cell swarms is the formation of an emergent vortex flow field in the frame of reference of the swarm (Fig. 5 *A* and *B* and *SI Appendix*, *Mathematical Modeling*, Section 1.4). In the stationary frame, this collective behavior equates to treadmilling-like dynamics, where cells at the top of the swarm tend to move toward the bacteria faster than the cells at the bottom. The cell flow profile increases quadratically from the floor to the swarm boundary (i.e. Poiseuille-like; Fig. 5 *A* and *B*) (30). These dynamics arise from the combined effect of a) the friction with the floor, which impedes directed cell motion near the floor, and b) the emergent swarm viscosity, which introduces correlations in the motion of neighboring cells. In other words, the model predicts that the emergent material properties of the swarm—mediated by the dynamic attachments and forces between cells and their physical environment—suppress the ability of cells at the swarm floor to respond to the gradient, giving rise to the vortex motion.

To experimentally test this form of collective cell motion, we tracked the 3D positions of individual cells from high spatial and temporal resolution live imaging of swarms (*SI Appendix*, Fig. S8 and Movies S8 and S9). To study the flow of cells, we averaged cell velocities across the face of the swarm (Fig. 5*C* and *SI Appendix*, Fig. S9 *A*–*C*). This analysis shows that cell motion within the swarm is indeed spatially organized as a vortex, where the magnitude of the average cell velocity at the top of the swarm is higher than for cells at the bottom (Fig. 5 *C* and *D* and *SI Appendix*, Fig. S10 *C* and *D*). Differences in cell motion along the vertical axes have a stronger relationship with distance from the swarm surface (depth) than from the floor (height) (Fig. 5 *C*′ and *C*″). During the traveling phase of swarm migration, cells at the swarm rear climb to the surface and cells at the swarm front move to the floor, with a cycle time of around 2 h along the outer swarm boundary (Fig. 5 *B* and *D*). This vortex cell motion persists during shedding, resulting in a larger contribution to clumps from cells at the swarm floor (Fig. 5*E*), indicating that the location of cells within the vortex influences the developmental program independently from the distance to the signaling gradient. Analysis of single cell motion across the swarm cross-section (*SI Appendix*, Fig. S9 *C* and *D*) shows that the mean speed of individual cells remains relatively constant throughout the swarm, with the exception of the swarm rear which tends to zero (*SI Appendix*, Fig. S9*E*). However, cell motion is uniform and directional at the swarm surface and, conversely, highly variable and predominantly misaligned with the gradient at the swarm core regions (*SI Appendix*, Fig. S10). In other words, the reason we observe a vortex flow is that although cells at the swarm core are just as motile as at the swarm surface, their motion is random, resulting in minimal net flow. In confirming model predictions, our results strongly suggest that the material properties of an active fluid—viscosity, friction, activity, and surface tension—emerge in large numbers of migrating cells, resulting in emergent flow fields that dictate cell organization.

## Discussion

Migration of groups of cells is a widespread feature of developmental and disease processes, from morphogenesis to wound healing to metastasis (31). Here, we have shown that the combined physics of surface tension, growth, and signal-directed activity are harnessed by migrating cell groups to pattern a cell population. Emergent fluid-like properties of the swarms cause clump shedding and vortex motion, which together have implications for how cells organize themselves in space, ultimately determining the decision of cells to remain in the undifferentiated state or to enter the developmental program. Our data—by validating our theory that the swarm behaves as an active droplet—suggest a mechanism of cell state allocation that differs from conventional models based purely on positional information (32) or cell-autonomous fate allocation (33) (Fig. 5*G*). The outcome for a single cell (to differentiate or proliferate) will depend on its location within the vortex at the time of droplet shedding. We refer to this mechanism of cell fate allocation as “musical chairs” decision-making. This is based on an analogy to the party game in which there are fewer chairs than children. When the music stops (droplet shedding), the circulating children scramble to find chairs—those not finding chairs are eliminated from the game (differentiation) while the other children get to stay in the game (with the continued possibility of feeding).

Our experiments and modeling explain how *Dictyostelium* collective cell shedding requires only a few biophysical processes: proliferation, chemotaxis, and intermittent physical cell–cell interactions. Our predictions are independent of the specific nature of the cell–cell interactions—previous theoretical work suggests that emergent fluid-like properties should, in general, arise in cell populations with attractive interactions between the cells (34, 35). We therefore expect that, although molecular details may vary substantially, similar physical mechanisms will be present in other developmental and disease contexts involving migration of groups of physically interacting cells in response to signaling gradients. Following this, we expect the shedding behavior and emergent flow profiles we have observed to be widespread across different tissue biology contexts.

Indeed, a system in which our model may have explanatory power is the migration of the vertebrate cranial neural crest (36). Collective migration here is mesenchymal, with all cells responding to the chemoattractant SDF1. Cells continually change their neighbors with vortex cell motion within cell groups (37). In addition, as with *Dictyostelium,* swarms of migrating neural crest cells show different shedding behaviors in different physical environments. Specifically, cell shedding is continuous in vivo, whereas explants migrating to SDF1 on fibronectin show shedding of cell clumps (38, 39), matching our observations of different cell shedding patterns for *Dictyostelium* migrating with varying degrees of resistance (Fig. 4). This similar spectrum of behavior suggests shared underlying physical properties, despite molecular differences between *Dictyostelium* and vertebrates in adhesion (40). Periodic shedding of cell clumps from migrating cell groups is also observed during lateral line formation (8, 9). Although these collective dynamics look morphologically similar to *Dictyostelium*, lateral line migration is likely to involve some different cell-level processes. For example, the advancing cell group shows limited neighbor exchange and the group has clear “leaders” and “followers.” In addition, chemical cues such as FGFs influence the behavior of the trailing zone of the group (41). A more relevant recent example implies active dewetting underlies aggregation of the mesenchymal cells to seed intestinal villi morphogenesis (42). In this context, clump formation seems to emerge from the acquisition of fluid-like behavior, rather than shedding from an existing fluid, but through the lens of active fluids, it will be useful to explore subsequent cell flows and their effects on villus expansion. This approach also seems relevant to understanding blood island formation, also characterized by mesenchymal cell aggregation, with application to describing the segregation of different cell types within the islands (43).

Tissue fluidity has emerged as an umbrella term to describe situations in which cells move relative to their neighbors. Our observations now reveal how cell–cell interactions during collective migration generate specific emergent fluid-like properties such as viscosity, surface tension, and spatially varying active stresses. The requirements for these fluid-like properties are easily satisfied in populations of migrating cells and we expect other cell and tissue contexts to also display features normally associated with basic physical systems such as dripping taps and raindrops sliding down windows. These fluid-like effects combine to determine different biological outcomes for cells within collectives and reveal how small perturbations in underlying biophysics can be harnessed by evolution to generate a broad diversity of morphological outcomes.

## Materials and Methods

### Cell Handling.

We used *Dictyostelium* AX2 cells with red fluorescent nuclei generated by insertion of a histone H2B-mCherry gene into the *act5* gene (44). Additional strains were used for comparison with AX2: the nonaxenic strain DdB (45) and the axenic strain, AX4 (46). For routine culturing, cells were inoculated on lawns of *Klebsiella* on SM agar (47) with washing steps in KK2 (20 mm KPO_4_ pH 6.0). To prepare feeding fronts for imaging, a *Klebsiella* suspension was spread onto diluted SM (1 SM: 4 KK2; 1.5% agar) before seeding a *Dictyostelium* colony with a concentrated drop of cells in KK2. For varying the bacterial concentration on plates, ratio of SM and KK2 was varied as required. For fluorescent imaging of bacteria, we used GFP-labeled *Klebsiella* (48). For heterogeneous cell labeling, AX2 H2B-mCherry cells were transformed with an extrachromosomal vector [pDM317(49)], which provides variable GFP expression. For generating *acaA* mutant cells, we replaced the hygromycin selection cassette in the *acaA* targeting vector, pPPI725 (11) with a blasticidin resistance cassette from pDM1079 (44) by swapping NheI/NotI fragments. Transformation, selection, and screening were carried out as described (50).

### Imaging.

For macrophotography, a Dino-Lite USB microscope was used to image feeding fronts (16). Samples were imaged every 2 min for 2 to 3 d, illuminating only during image capture. To prevent desiccation, samples were imaged in a custom-built humidified chamber. Macrophotography imaging data were analyzed manually.

To 3D image both bacteria and *Dictyostelium* cells across feeding fronts, we used an Intelligent Imaging Innovations (3i) Marianas Lightsheet microscope (dual inverted selective plane illumination microscope, diSPIM) (51). Imaging was carried out from above the sample at 45° to the surface with oil-dipping 10× objectives, using 3i’s software SlideBook in single sided illumination mode. Samples were submerged in silicone oil which has high levels of dissolved oxygen and prevents desiccation (52). See *SI Appendix* for more detailed imaging protocols. Raw imaging data at different spatiotemporal scales can be accessed at the following link: https://doi.org/10.6019/S-BSST1979 (53).

### Image Analysis.

Swarm shape was quantified using Matlab’s edge detection algorithm to identify the upper and lower surfaces of binarized images of the *Dictyostelium* and bacteria populations. The quantity of bacteria was estimated by a sum z-projection. The location of the swarm front and the rear were defined as the positions where the swarm height was 30 µm. The bacteria gradient was estimated by the spatial derivative of the total amount of bacteria across a distance of 6 cell widths. The mean and minimum values of the bacteria gradient were calculated as the mean and minimum values between swarm peak and rear.

Bacterial flow fields were quantified using PIV (PIVlab) applied to the bacteria and cell nuclei channels of each 2D plane (parallel to the direction of swarm travel) and then spatially averaged at each time point. Cell flow fields within the swarm were quantified by tracking (TrackMate: simple LAP tracker, CSVImporter) the centroids of nuclei (masked via watershed segmentation; SCF-MPI-CBG). Single-cell velocities were determined by the second-order central finite difference of cell positions. The mean cell velocity field across the swarm was calculated by averaging the velocity of each cell relative to the peak of the swarm, averaged over a 15-min period. See *SI Appendix* for more detailed analysis protocols.

## Supplementary Material

Appendix 01 (PDF)

Movie S1. Pattern formation during feeding front expansion (relates to Fig. 1A). Macrophotography time-lapse of wild type (top) and acaA- (bottom) *Dictyostelium* colonies. The feeding front appears as a ring that expands into the surrounding bacterial field. Cell clumps appear as circles that shed from the localised areas of swarm elongation. The *Dictyostelium* developmental programme is organised by cAMP signaling is seen initially as a streaming pattern. Scale bars: 2mm.

Movie S2. Cell clump shedding dynamics (relates to Fig. 1A′). Same as Movie S1 but showing a different dataset obtained at a higher frame rate with a smaller field of view. Video shows the coursening of an irregularly shaped cell clump into two circular clumps, that persist while surrounding isolated cells progress through the *Dictyostelium* developmental programme: formation of cell streams, tipped-mounds, migrating slugs, and eventually fruiting bodies. Scale bars: 500 μm.

Movie S3. Dynamics of cell clump shedding and gradient remodelling – top view (relates to Fig. 1B). Maximum projection (birds’-eye-view) of light sheet imaging of feeding front dynamics showing penetration into the bacteria field and shedding of cell clumps. The cell nuclei are labelled in orange and the bacteria are labelled in green. Scale bar: 200μm.

Movie S4. Self-generated gradient dynamics – side view (relates to Fig. 1C). Maximum projection (top panel: side-view, bottom panel: birds’-eye-view) similar to Movie S3 but with a different data set obtained at a higher frame rate with a smaller field of view. Cell nuclei are labelled in orange and bacteria are labelled in green. Scale bar: 100μm.

Movie S5. Quantification of swarm height and bacteria quantity (relates to Fig. 2A). Quantification of the swarm height (top) and bacteria quantity (bottom) of the data shown in Movie S3. The tick units are in μm. See figure legend for Fig 2A.

Movie S6. Model simulation. Simulation of the active thin film model of directed swarm migration. Top: model predictions for swarm dynamics during shedding in the stationary reference frame of the lab. Colour map indicates the magnitude of the horizontal cell velocity. As in Fig. 3B, black dots indicate respectively the front on rear of the swarm. Left: simulated swarm dynamics during shedding in the co-moving reference frame of the swarm front. Colour map indicates the magnitude of the horizontal cell velocity in the co-moving reference frame. Right: simulated swarm dynamics and chemoattractant profile (dark red line) during shedding in the co-moving reference frame of the swarm front. Also shown in dark red is the region where the gradient in the chemoattractant drops below a cell sensitivity threshold (SI Appendix, Mathematical Modeling).

Movie S7. Periodic shedding of cell clumpes along lines of bacteria (relates to Fig 3C). Macrophotography time lapse of wild type *Dictyostelium* cells migrating along thin lines of bacteria, resulting in the periodic shedding of cell clumps (1 shedding event per 4.35h). Scale bar: 1mm.

Movie S8. High resolution imaging of the cell nuclei (relates to Fig. 4). Cells at the top of the swarm move in a more directional manner than cells at the floor. Same as Movie S2, but showing a different dataset obtained at a higher frame rate and a smaller field of view for cell tracking, without imaging the bacteria. Scale bar: 100μm.

Movie S9. Imaging cell flows in heterogeneously-labeled swarms. Cells at the top of the swarm move in more directional manner than cells at the floor. Related to Movie S8, but showing a different dataset obtained using cells heterogeneously labelled with GFP, to enable visual inspection of single cell flows. Movie length: 1hr. Tick spacing: 50μm.

## Data Availability

Time-lapse 3D images data have been deposited in Biostudies (https://doi.org/10.6019/S-BSST1979) (53). Code for modelling is available at https://github.com/giuliacelora/Dictyostelium-Swarm-Migration (54).
