# Supplementary material for: Pattern formation along signaling gradients driven by active droplet behavior of cell swarms
Source: Proc Natl Acad Sci U S A. 2025 May 20;122(21):e2419152122. doi: 10.1073/pnas.2419152122 (PMC12130873; doi:10.1073/pnas.2419152122)
Supplement: Supplementary file 1 — Appendix 01 (PDF) [file pnas.2419152122.sapp.pdf]

Supporting Information for

Pattern formation along signaling gradients driven by active droplet behavior of cell swarms

Hugh Z Ford, Giulia L Celora, Elizabeth R Westbrook, Mohit P Dalwadi, Benjamin J Walker, Hella Baughmann, Cornelis J. Weijer, Philip Pearce, Jonathan R Chubb

Philip Pearce; Jonathan R Chubb.
E-mail: philip.pearce@ucl.ac.uk; j.chubb@ucl.ac.uk

This PDF file includes:

Figs. S1 to S10
Legends for Movies S1 to S9
SI Appendices: (1) Materials and Methods, (2) Mathematical Modeling
Figs. SM1 to SM8
Tables SM1 to SM2
SI References

Other supporting materials for this manuscript include the following:

Movies S1 to S9

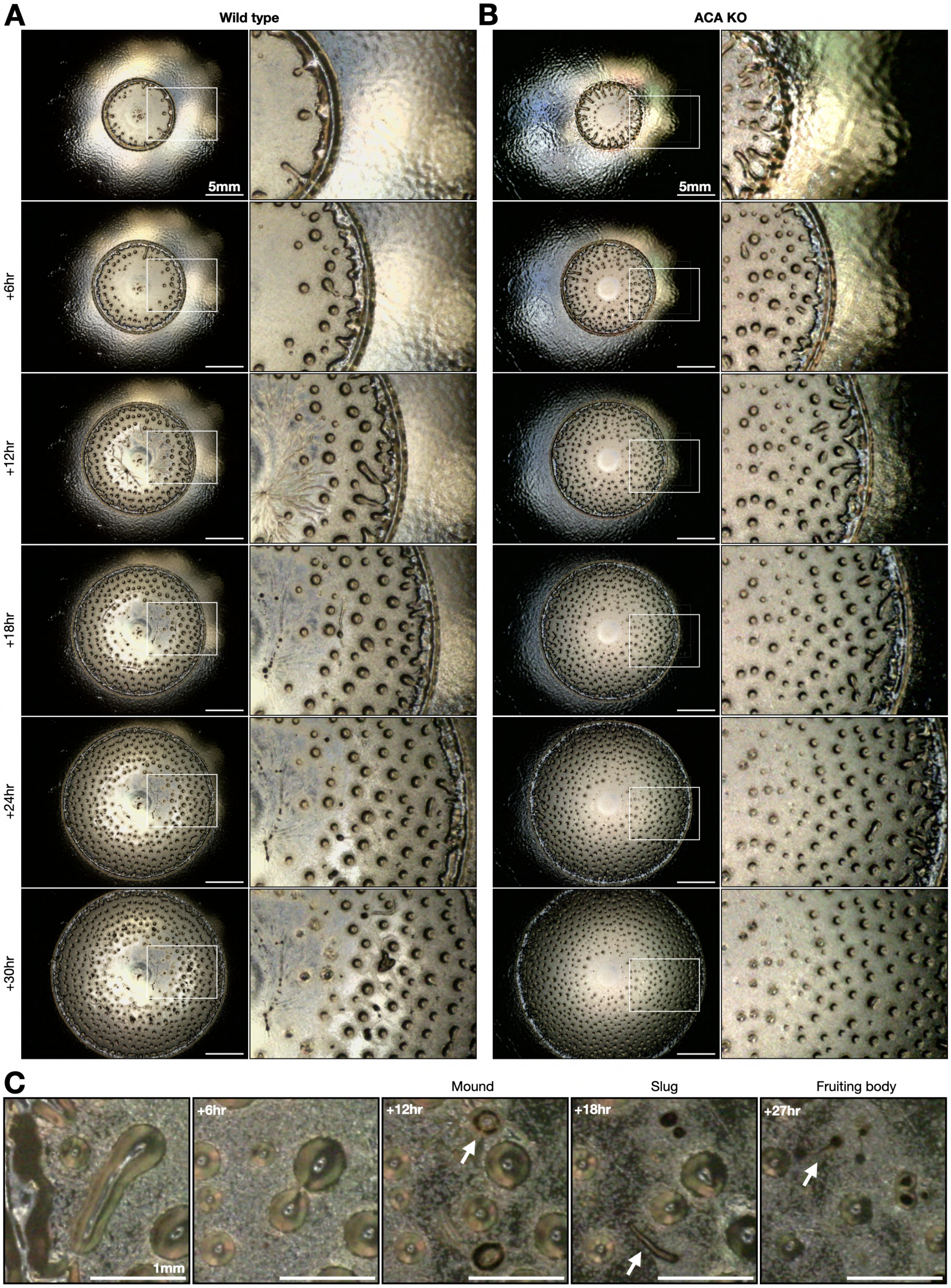

Fig. S1. Patterns of cell clump shedding behind expanding feeding fronts. (A) Macrophotography of feeding fronts at 6h intervals, showing feeding front progression, patterns of clump deposition and the onset of later stage development (*e.g.*, see stream formation in the 12h panel). Scale bar 5mm. (B) Feeding fronts of acaA- mutants showing similar feeding front progression and clump deposition but no later stage development. (C) Close-up of wild-type cell clump progression, showing an irregularly shaped clump pinching off the front, then coarsening into two clumps, which disperse after a day or so. The clumps are persistent despite the occurrence of late-stage development of surrounding cells (arrows).

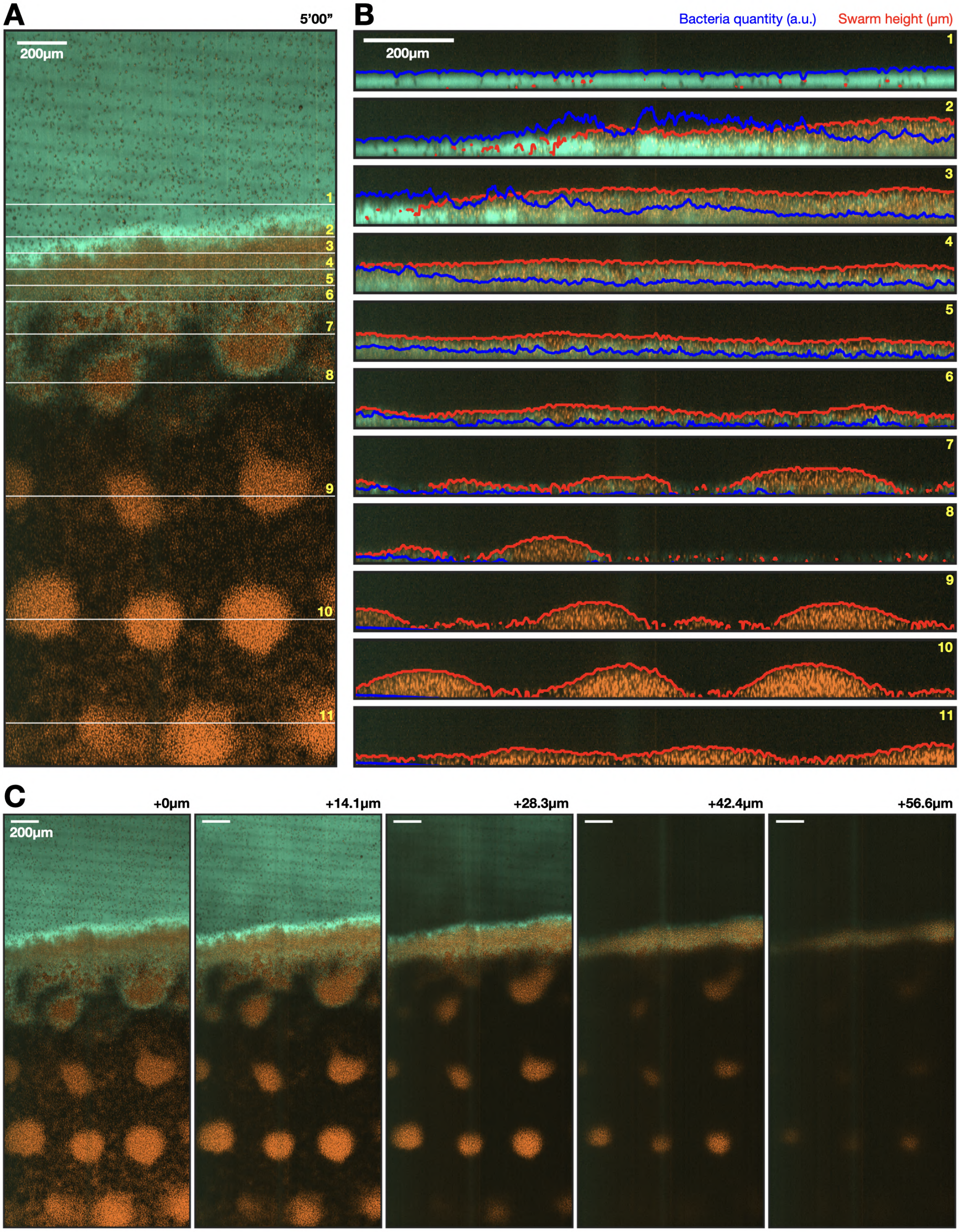

Fig. S2. Quantification of swarm height and bacteria quantity from light sheet data. (A) Light-sheet imaging of *Dictyostelium* feeding fronts showing cell nuclei (orange) and bacteria (green). Shown is a single birds'-eye-view of the entire imaging window. (B) Side-view cross sections of the same data in A showing the measured swarm boundary (red line) and quantification of the bacteria (blue line). Each section corresponds to a line in panel A. (C) Same data as A, but showing sectioning out of the plane.

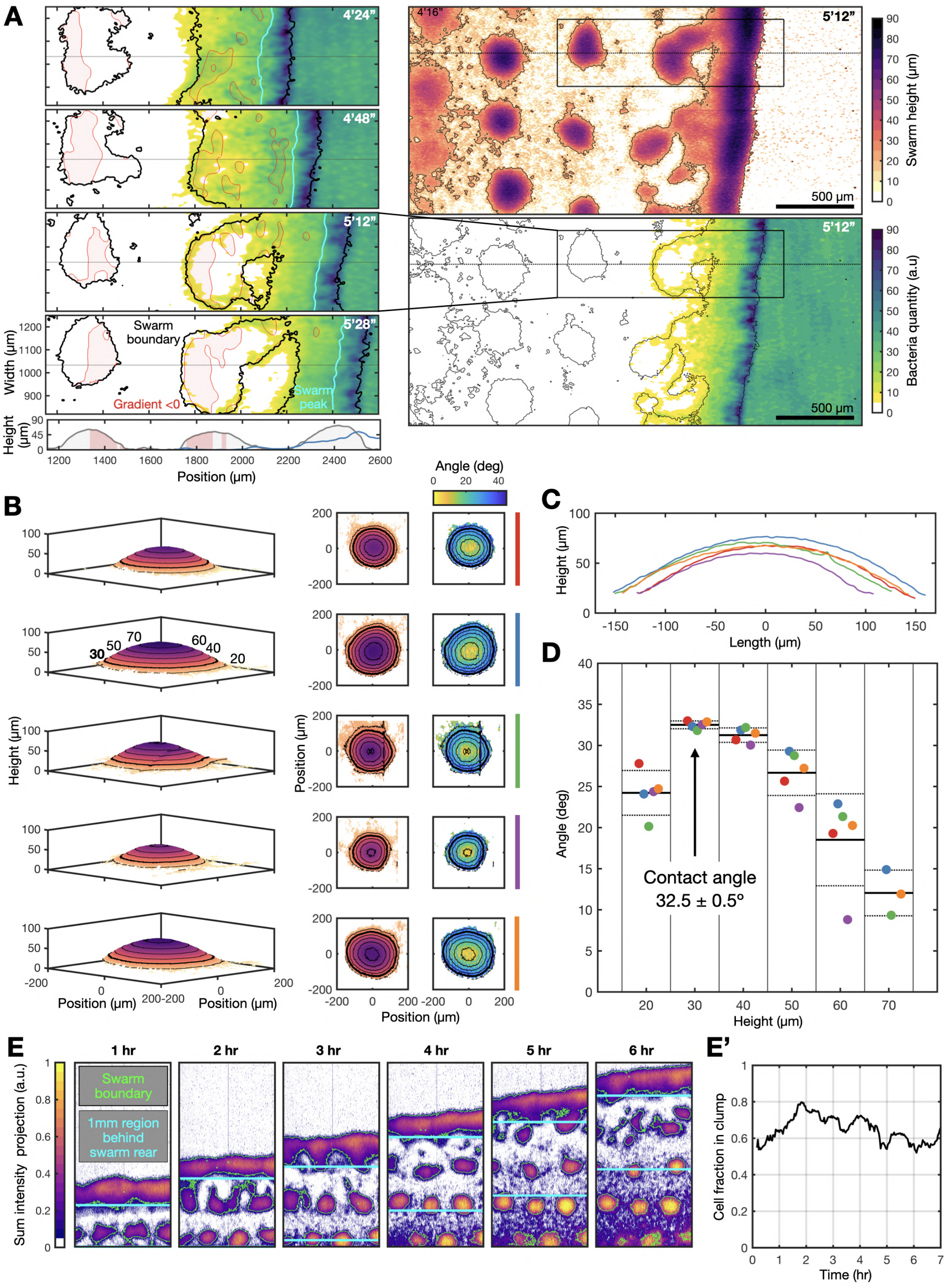

Fig. S3. Quantitative features of cell clumps. (A) Quantification of the cell swarm height and bacteria quantity from the data shown in Fig. 1B. The left column shows the distribution of bacteria throughout a shedding event, while highlighting the swarm boundary (where the height is 30μm) and peak and where the bacteria gradient is flattened (value less than zero). Right panels show the swarm height and bacterial quantity from the entire field of view. These data are heatmaps of the data in Fig. 2A. (B) 3D plots, with contours, of the surface of five cell clumps (average over 60 min of imaging) from the data shown in Fig. 1B. Also shown is the angle of the surface gradient (right column). (C) Cross sections of each clump in B, shown in different colours. (D) Scatter plots of the average angle of the surface gradient for each clump (colour), for different heights on the clump surface (x-axis). Also shown is the mean (thick line) and standard deviation (dotted line) for each height. The smallest height (30μm) at which the variance is negligible is highlighted and estimated as $32°$-$33°$. (E) Proportions of cells in clumps and isolated cells. Sum intensity projections (cell nuclei) of the 3D images shown in Fig. 1B and the fraction of cells that are left behind the feeding front within a clump as opposed to as single cells. (E') This value was quantified by comparing the total nuclei intensity within and outside clump boundaries (contours where the population height is 30μm, highlighted in green) in a 1mm region behind the swarm rear (highlighted in cyan).

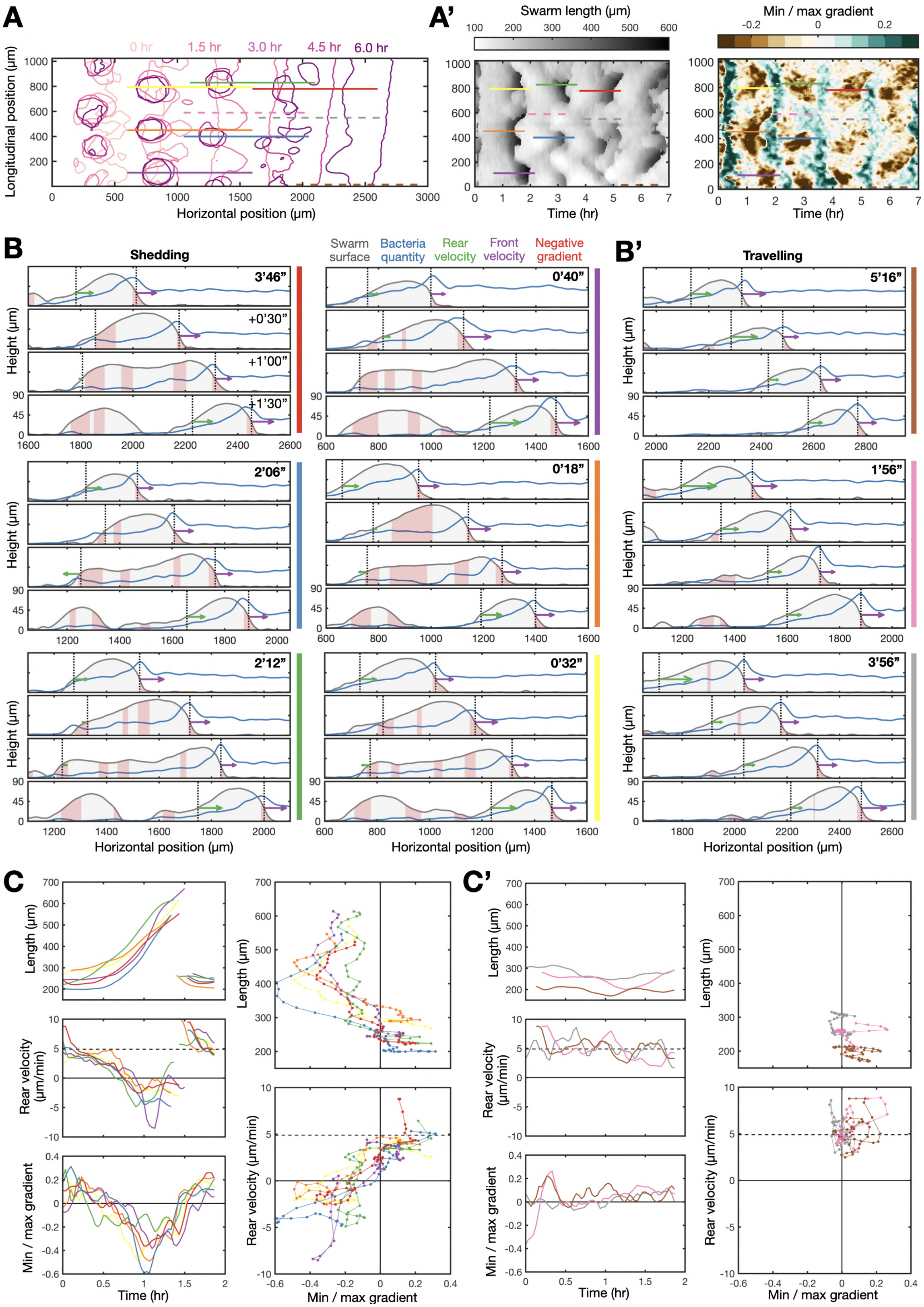

Fig. S4. Swarm shape dynamics during travelling and shedding. (A) Contours of swarm and clump boundaries shown at 1.5hr intervals (colour). (A') Swarm length (left) and the ratio of the minimum and maximum gradient within the swarm (right) as a function of position across the imaging window and time. Coloured lines indicate the spatial and time points for the representative data shown in B. (B) Cross sections of a swarm over a 90 min period during the shedding phase (elongation and splitting). (B') Cross sections of a swarm over a 90 min period during the travelling phase (no elongation). The colour bands refer to colours in A. These plots are the same representation as Fig. 2B, using a different sample point of the same dataset. (C) Elongation associated with a loss of positional information from the front due to a negative minimal gradient. Plots of the swarm length (top left), rear velocity (middle left) and the ratio of the minimum and maximum gradient (bottom) for the swarm cross sections shown in B (shedding phase). Also shown is the swarm length (top right) and rear velocity (bottom right) plotted against the ratio of the minimum and maximum gradient. The dashed lines indicate the mean swarm speed. (C') Same as C, but for the cross section shown in B' (travelling phase).

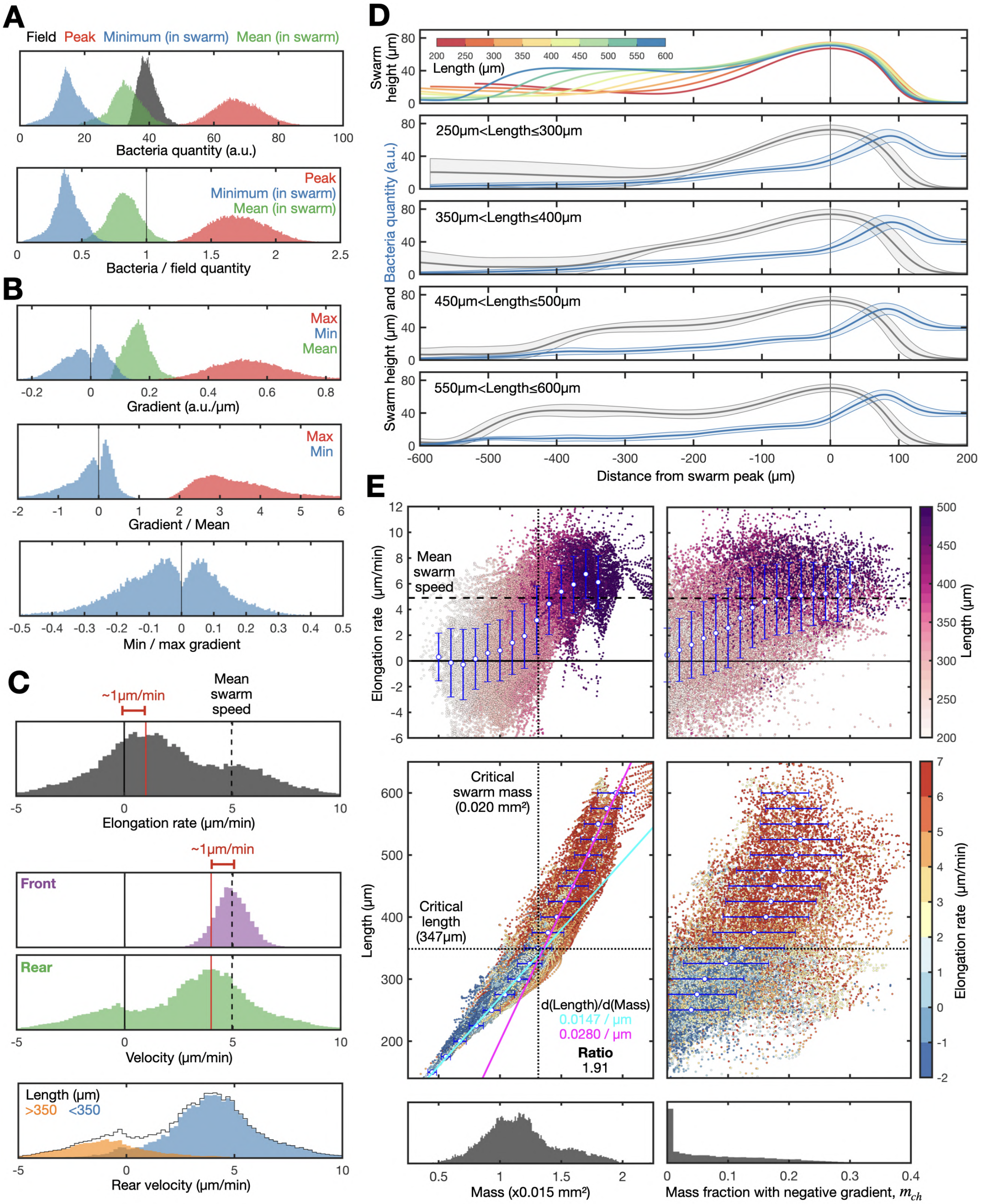

Fig. S5. Extended analysis of cell swarm and signal gradient interactions. (A) Histograms of the minimum (blue) and mean (green) quantity of bacteria within the swarm boundary, and the quantity of bacteria at the bacteria peak (red) and bacteria field (black - averaged across 100μm-200μm ahead of the bacteria peak). Also shown are the minimum, mean and peak bacteria quantity relative to the quantity of the bacteria field (bottom). (B) Gradient statistics: histograms of maximum (red), minimum (blue, neglecting the minimum gradient at the front of the bacteria peak) and mean (green) value of the gradient within the swarm (top), the ratio of the maximum and minimum gradient to the mean gradient (middle), and the ratio of the minimum gradient and maximum gradient (bottom). The maximum value (at the front of the swarm) is consistently greater than twice the mean value of the gradient within the swarm. The minimum gradient is double-peaked around zero. Overall, this indicates the gradient at the front is consistently positive, whereas the gradient in the swarm fluctuates. (C) Histogram of the elongation rate (black), showing two elongation states: slow and fast. Also shown are the front (purple) and rear (green) velocities, highlighting the mean swarm speed (dashed line) revealing the difference in the mean and rear swarm velocity- which accounts for the low baseline elongation. Bottom panel: histograms of the rear swarm velocity for regions where the swarm length is less than (blue) and greater (orange) than the critical swarm length (350μm) separating the travelling and shedding phases of swarm behaviour, shown in Fig. 2F. (D) Low variance swarm and bacterial profiles at different swarm lengths, showing reproducible swarm dynamics. Top: mean swarm boundaries (taken from all spatial and time points) for swarms of different lengths (colour). Bottom: mean and standard deviation of the swarm boundaries (grey) and bacteria quantity (blue) for swarms of different lengths. (E) Comparing swarm dynamics, size and mass (area under the curve) with model predictions in Fig. 3F. Left panels show how elongation rate (top), swarm length (middle), relate to the mass (bottom). Below a critical mass, the length linearly increases (cyan line) with mass. Above this critical mass, the length (and elongation rate) increases at a faster rate (around twice as fast). Critical values of both mass (0.020 mm^2) and length (347μm) were derived from the intersections between the linear fits. Right panels show the same analysis, comparing length and elongation with respect to the mass fraction of the swarm that experiences a negative gradient. A steady increase in the elongation rate (top) and length (middle) occurs as the mass fraction with a negative gradient increases. Error bars (top) are SD of elongation rates. Error bars (middle panels) are SD of swarm mass/mass fraction.

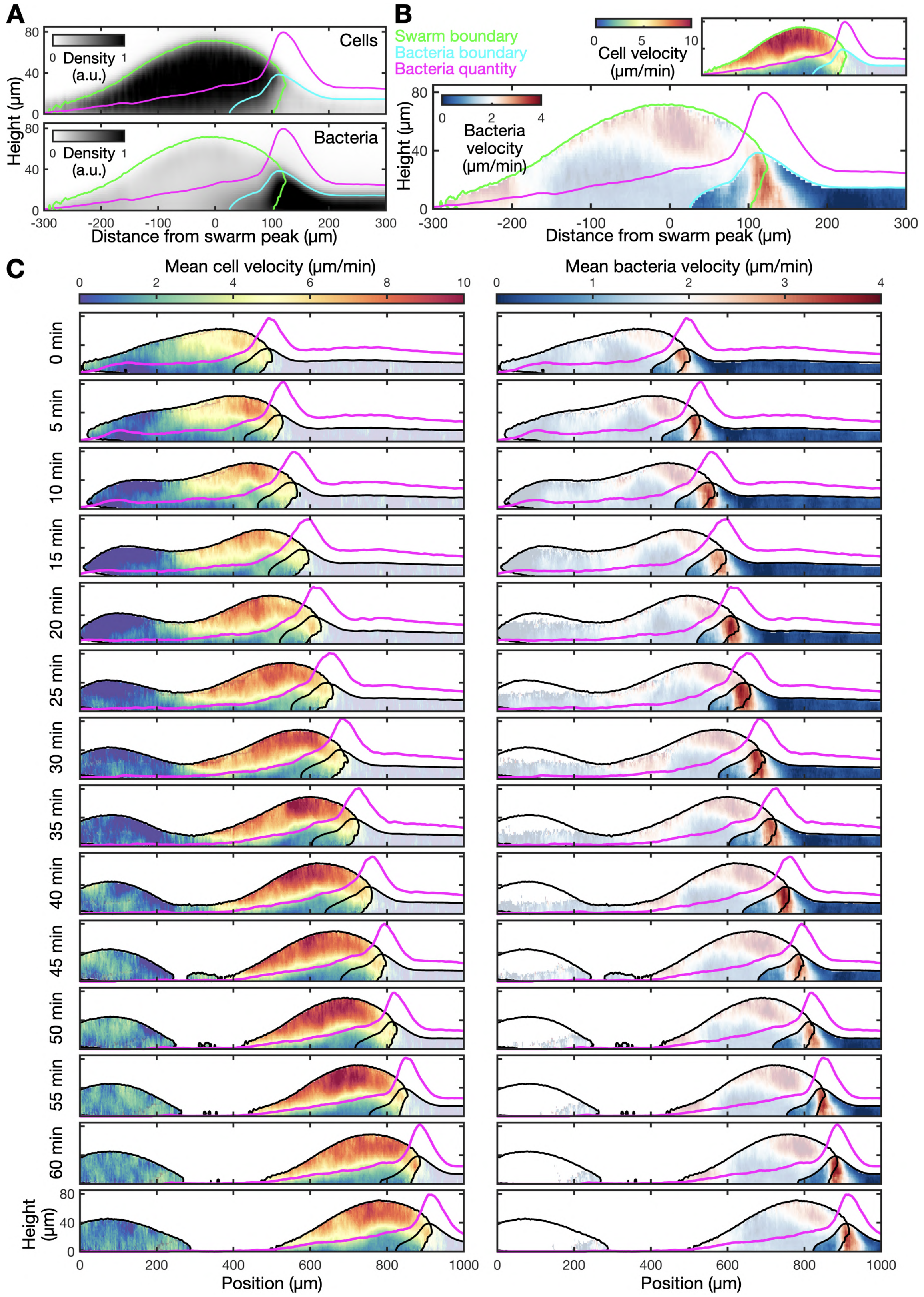

Fig. S6. The "snowplough" model: particle image velocimetry (PIV) of cell nuclei and bacteria. (A) Mean density of cells (top) and bacteria (bottom) across the face of the swarm (dataset shown in Fig. 1C). Also shown are the boundaries of the swarm (green) and bacteria (blue), and the total sum of the bacteria along the swarm length (pink). (B) Velocity of bacteria is maximal at the leading edge of the swarm. Plots show the velocity fields of bacteria and cells across the face of the swarm measured using PIV. (C) Velocity fields of bacteria and cells over a 1h period during a shedding event.

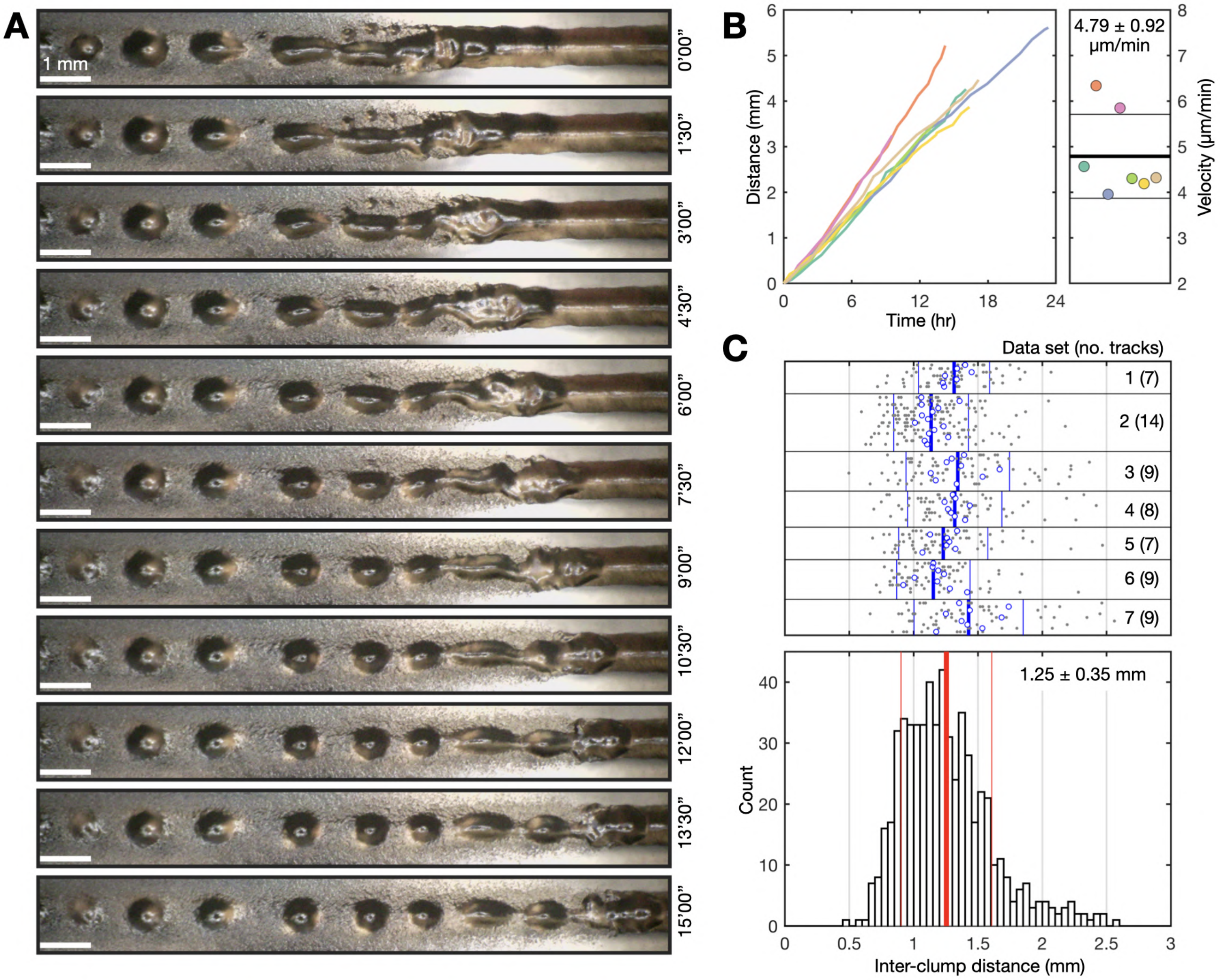

Fig. S7. Estimating the periodic shedding rate of cell clumps. The shedding rate (1 clump per 4.35hr) was estimated by dividing the swarm speed by the interclump distance of swarms on thin bacterial lines. (A) Time-lapse macrophotography of an isolated and compact *Dictyostelium* swarm travelling along, and clearing, a thin line ($\sim 500\mu$m width) of bacteria. (B) Tracks of the position (left) and average speed (right) of *Dictyostelium* swarms travelling along bacteria lines (representative data shown in A). Seven biological repeats are shown in colour. (C) Quantification of the average distance between cell clumps generated by swarms travelling along bacterial lines. The experiments were performed by generating a set of bacterial lines on a single plate. 7 experiments are shown, 1 per box. Each grey dot within a box is the distance between clumps, with the mean (blue dots) of the inter-clump distance of individual lines, together with the mean (thick blue line) and standard deviation (thin blue line) for each experiment. The bottom panel shows a histogram of all measured inter-clump distances, together with the aggregated mean (thick red line) and standard deviation (thin red line).

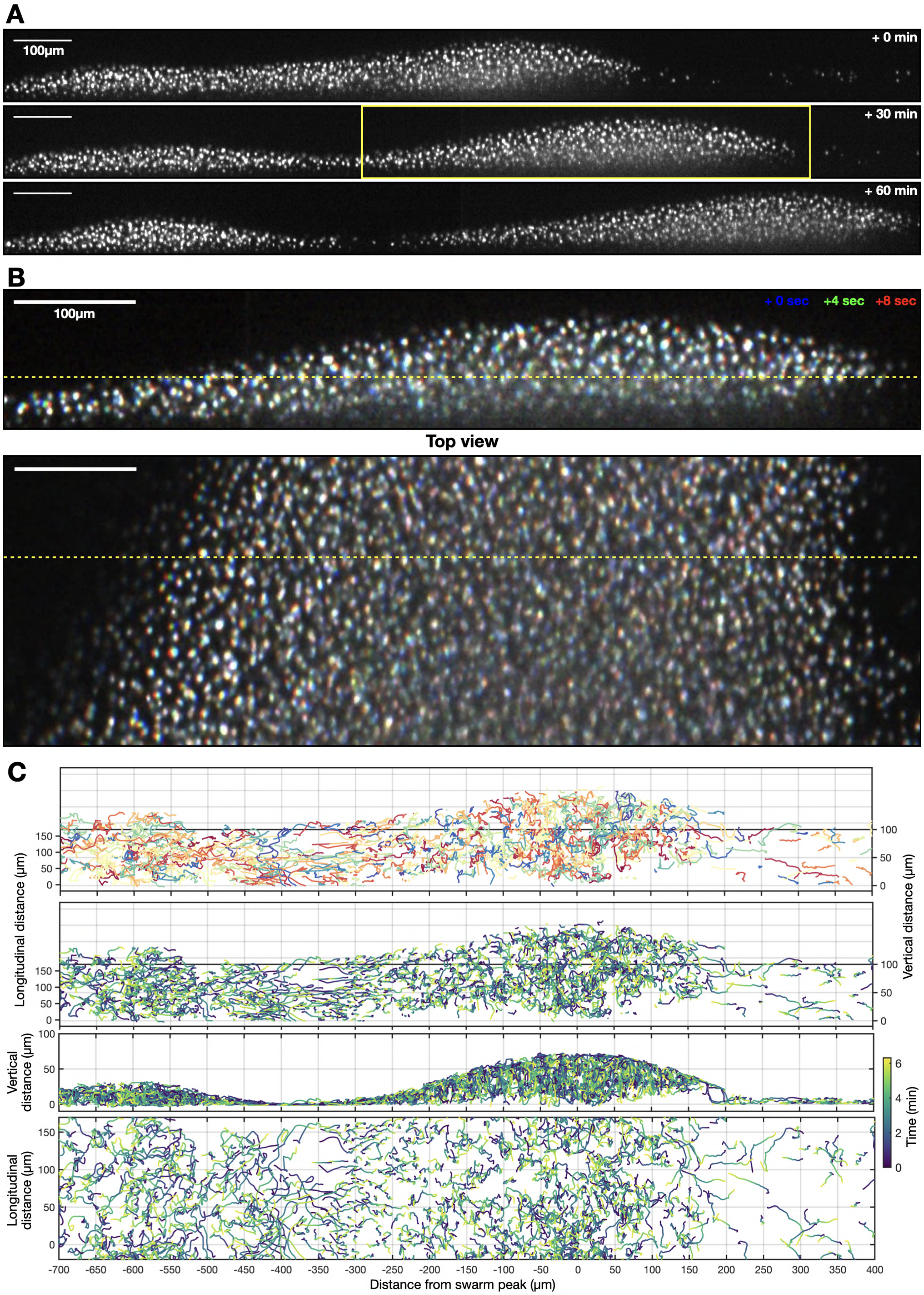

Fig. S8. Tracking cell motion from high spatiotemporal resolution imaging of the swarm. (A) Cross-section of the swarm (raw data) every 30 min during a shedding event. (B) Side and top views of the region of the swarm from the yellow box in A with three overlaid time points, captured at 4s intervals. Different time points are shown in different colours. The dashed yellow line in the top panel shows the vertical slice displayed in the bottom panel (and vice versa). (C) A sub-sample of 3D cell tracks coloured by their cell ID (top panel) and time (bottom three panels). Bottom three plots show (from top to bottom) the same 3D tracks from an alternative viewpoint (at a 45 degree angle) from the side and from the top.

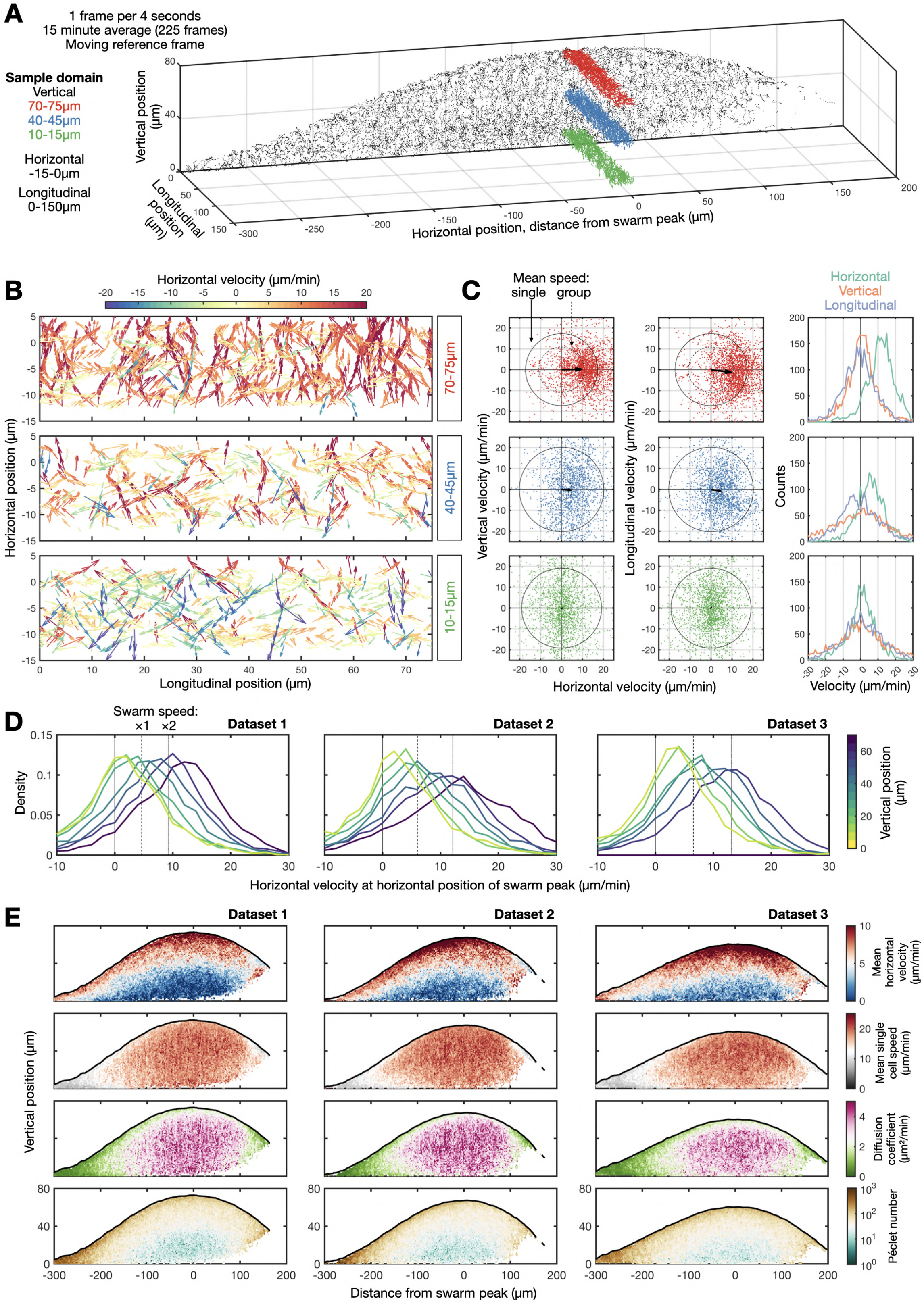

Fig. S9. Quantification of cell motion within the swarm: finding the balance between advection and random motion. (A) 3D section of the swarm. In grey are individual cell coordinates from a thin slice of the swarm. The red, blue and green vectors show all measured velocities of cells at small longitudinal sections across the 3D volume. (B) Zooming in on the three coloured slices in A to show velocity vectors. (C) Scatter plots of the 3D velocities measured at the three different swarm heights. Black arrows indicate average motion. Solid circles represent the average speed of single cells, which is relatively invariant. Dotted circles are the average speed of the group at that swarm position, which changed depending on swarm's vertical position. Right panels show the distributions of the horizontal, vertical and longitudinal velocities at the three different locations. (D) Transition from directed to random motion at various vertical positions in the swarms. These plots (from three biological repeats) show how the horizontal velocity (green curve in right panel of C) changes as a function of the vertical position in the swarm. At the top of the swarm, cells move at twice the swarm speed. At the bottom, their average horizontal

velocity is close to zero. (E) Top panels show the mean horizontal velocity across the swarm face. 2nd row shows the mean single cell speed. 3rd row shows the diffusion coefficient and 4th row shows the Péclet number. Data shown for 3 biological repeats. Overall, cell motion is uniform and directional at the swarm surface and, conversely, highly variable and diffusive at the swarm core regions. This transition between order and disorder is summarised by a steady reduction from large Péclet numbers at the swarm surface, indicative of strongly directed motion, to a Péclet number of ≈ 1 at the swarm core, indicative of a balance between directed and random motion. Overall, these results imply that the reduction in average cell movement at the swarm core is caused by a transition from advective- to diffusive-dominant motion.

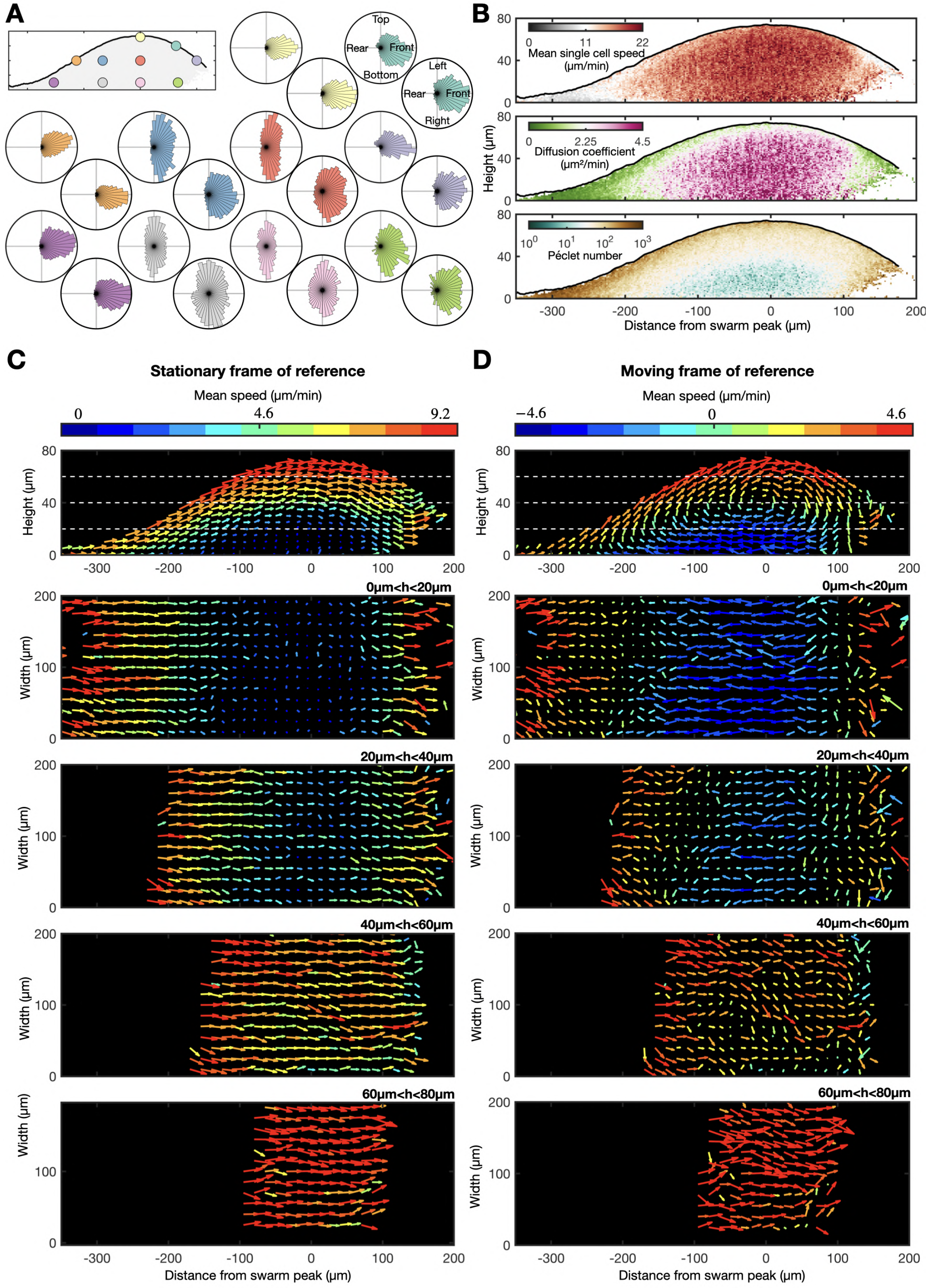

Fig. S10. Quantification of cell motion within the swarm. (A) Polar distributions of cell orientations at various points across the face of the swarm (inset panel). Each position (colour) has two plots showing the 2D planes of the side and top views. (B) The distribution of the mean speed of individual cells (top), the diffusion coefficient (middle) and the Peclet number (bottom). The Peclet number reflects the ratio of directed (advective transport) to random (diffusive transport) cell motion. If the number is much larger than 1, motion is predominantly directed. If the number is less than 1, motion is predominantly random. (C) Mean cell velocity fields across the swarm (as in Figure 5D), in addition to 4 sections at different swarm heights, in the stationary (observer) reference frame. Arrows are coloured by the velocity magnitude. The stationary reference frame shows treadmilling-like behaviour. (D) Same as C, but showing the moving reference frame, revealing vortex cell flows. In both C and D, there is no substantial flow perpendicular to the direction of swarm travel. The lateral motion apparent at swarm core in A is averaged out by uncorrelated motion of cells, as indicated by the diffusion field shown in B.

Movie S1. Pattern formation during feeding front expansion (relates to Fig. 1A). Macrophotography time-lapse of wild type (top) and acaA- (bottom) *Dictyostelium* colonies. The feeding front appears as a ring that expands into the surrounding bacterial field. Cell clumps appear as circles that shed from the localised areas of swarm elongation. The *Dictyostelium* developmental programme is organised by cAMP signaling is seen initially as a streaming pattern. Scale bars: 2mm.

Movie S2. Cell clump shedding dynamics (relates to Fig. 1A′). Same as Movie S1 but showing a different dataset obtained at a higher frame rate with a smaller field of view. Video shows the coursening of an irregularly shaped cell clump into two circular clumps, that persist while surrounding isolated cells progress through the *Dictyostelium* developmental programme: formation of cell streams, tipped-mounds, migrating slugs, and eventually fruiting bodies. Scale bars: $500\,\mu$m.

Movie S3. Dynamics of cell clump shedding and gradient remodelling – top view (relates to Fig. 1B). Maximum projection (birds'-eye-view) of light sheet imaging of feeding front dynamics showing penetration into the bacteria field and shedding of cell clumps. The cell nuclei are labelled in orange and the bacteria are labelled in green. Scale bar: 200μm.

Movie S4. Self-generated gradient dynamics – side view (relates to Fig. 1C). Maximum projection (top panel: side-view, bottom panel: birds'-eye-view) similar to Movie S3 but with a different data set obtained at a higher frame rate with a smaller field of view. Cell nuclei are labelled in orange and bacteria are labelled in green. Scale bar: 100μm.

Movie S5. Quantification of swarm height and bacteria quantity (relates to Fig. 2A). Quantification of the swarm height (top) and bacteria quantity (bottom) of the data shown in Movie S3. The tick units are in μm. See figure legend for Fig 2A.

Movie S6. Model simulation. Simulation of the active thin film model of directed swarm migration. Top: model predictions for swarm dynamics during shedding in the stationary reference frame of the lab. Colour map indicates the magnitude of the horizontal cell velocity. As in Fig. 3B, black dots indicate respectively the front on rear of the swarm. Left: simulated swarm dynamics during shedding in the co-moving reference frame of the swarm front. Colour map indicates the magnitude of the horizontal cell velocity in the co-moving reference frame. Right: simulated swarm dynamics and chemoattractant profile (dark red line) during shedding in the co-moving reference frame of the swarm front. Also shown in dark red is the region where the gradient in the chemoattractant drops below a cell sensitivity threshold (SI Appendix, Mathematical Modeling).

Movie S7. Periodic shedding of cell clumpes along lines of bacteria (relates to Fig 3C). Macrophotography time lapse of wild type *Dictyostelium* cells migrating along thin lines of bacteria, resulting in the periodic shedding of cell clumps (1 shedding event per 4.35h). Scale bar: 1mm.

Movie S8. High resolution imaging of the cell nuclei (relates to Fig. 4). Cells at the top of the swarm move in a more directional manner than cells at the floor. Same as Movie S2, but showing a different dataset obtained at a higher frame rate and a smaller field of view for cell tracking, without imaging the bacteria. Scale bar: 100μm.

Movie S9. Imaging cell flows in heterogeneously-labeled swarms. Cells at the top of the swarm move in more directional manner than cells at the floor. Related to Movie S8, but showing a different dataset obtained using cells heterogeneously labelled with GFP, to enable visual inspection of single cell flows. Movie length: 1hr. Tick spacing: 50μm.

Supporting Information Text

1. Materials and Methods

Cell handling. We used *Dictyostelium* AX2 cells with red fluorescent nuclei generated by insertion of a histone H2B-mCherry gene into the act5 gene (1). For routine culturing, cells were innoculated on lawns of Klebsiella on SM agar (2) with washing steps in KK2 (20mm KPO4 pH 6.0). To prepare feeding fronts for imaging, 120μl of a Klebsiella suspension was evenly spread across a 9cm agar plate containing diluted SM (1 SM: 4 KK2; 1.5% agar) left to almost completely dry before seeding a *Dictyostelium* colony. Care was taken to generate an even and smooth bacterial lawn. For varying the bacterial concentration on plates,the ratio of SM and KK2 was varied as required. Two additional strains were used for comparison with AX2: the non-axenic strain DdB (3) and the other standard strain, AX4 (4). For fluorescent imaging of bacteria, we used GFP- labelled Klebsiella (5). *Dictyostelium* colonies were seeded by resuspending around 107 cells from the leading edge of an initial SM colony into 0.1mL of KK2 buffer. 1μl of this suspension was spotted onto the centre of the bacterial lawn. To prepare lines of bacteria, we painted strips of bacteria in rows on SM plates. *Dictyostelium* cells were then spotted at the base of the lines of bacteria. For heterogeneous labelling of the AX2 H2B-mCherry cells, these were transformed with an extrachromosomal vector expressing GFP (pDM317 (6)), which provides variable GFP expression. For generating the acaA mutant cell lines, we replaced the hygromycin selection cassette in the acaA targeting vector, pPPI725 (7) with a blasticidin resistance cassette from pDM1079 (1) by swapping NheI/NotI fragments. The targeting vector was linearised for transformation with NgoMIV. Transformation, selection and screening were carried out as described (8).

Live cell imaging. For macrophotography, a Dino-Lite USB microscope was used to image feeding fronts of *Dictyostelium* (1-2 days after inoculation) at 22°C (9). The sample was imaged every 2 minutes for 2-3 days, illuminating the sample only during image acquisition. To prevent desiccation, samples were imaged in a custom-built humid chamber – a completely dark and enclosed box, except for a hole at the top for imaging, with a platform (sample mounting) surrounded by a water reservoir. Macrophotography imaging data was analysed manually. To 3D live image both bacteria and *Dictyostelium* cells across the feeding fronts of *Dictyostelium*, we used a 3i Marianas light-sheet microscope (Dual Inverted Selective Plane Illumination Microscope, diSPIM) (10). Illumination and imaging were carried out above the sample at 45° to the surface with oil-dipping 10x objectives. Samples were submerged in silicone oil which has high levels of dissolved oxygen and prevents dessication (11). As we observed the same clump deposition dynamics by light sheet and macrophotography (no oil), we were satisfied that the samples were not subject to disruptive effects of immersion (*e.g.*, hypoxia). Imaging data were collected at 3 different spatiotemporal scales. Data in Figs. 1B & 2, Figs. S2–S5 and Movies 3 and 5 were obtained by moving the sample 3mm through the light sheet along the axis parallel to feeding front travel at 2μm step sizes every 2 minutes, imaging with both red (nuclei) and green (bacteria) light. The total volume of the field-of-view was 3000μm x 1300μm x 200μm with voxel dimensions 1.3μm x 1.3μm x 2μm (width x length x height). Data in Fig. 5, Figs. S8 and S9 and Movie 8 were obtained by moving the sample 150μm downwards through the light sheet at 1μm step sizes every 4 seconds, imaging just the red nuclei. The data presented in Fig. 1C, Fig. S6 and Movies 4 and 9 were obtained by moving the sample 500μm through the light sheet, perpendicular to the direction of swarm travel, at 1μm step sizes every 15 seconds, imaging both green and red channels. The total volume of the field-of-view was 150μm x 1300μm x 200μm with voxel dimensions 1.3μm x 1.3μm x 2μm (width x length x height). Slidebook2022 was used to deskew the imaging data and export to TIFF format for downstream analysis. Raw imaging data at the different spatial scales can be accessed at https://doi.org/10.6019/S-BSST1979.

Image analysis. To quantify swarm shape, the upper and lower surfaces of both the *Dictyostelium* and bacteria populations were calculated using Matlab's edge detection algorithm applied to binarised images of the cross-sectional plane perpendicular and parallel to the direction of travel. The surface of the agar was determined at each time point by fitting a plane to the bottom surfaces of the bacteria and *Dictyostelium* populations. The quantity of bacteria was estimated by a sum z-projection. The justification for using bacterial quantity rather than height is that bacteria are dispersed across the mound and, unlike for the cell nuclei, the bacterial height would poorly reflect the amount of bacteria. The location of the swarm front and the rear were defined as the positions where the swarm height was 30μm. The bacteria gradient was estimated by the spatial derivative of the total amount of bacteria across a distance of 6 cell widths (6 x 13μm). The mean and minimum values of the bacteria gradient were calculated as the mean and minimum values between the swarm peak and the rear.

To estimate the flow field of bacteria (Fig. S6), particle image velocimetry (using PIVlab, Matlab) was applied to the bacteria (green) and cell nuclei (red) channels of each 2D plane (parallel to the direction of swarm travel) and then averaged (500μm) at each time point.

To estimate cell flow fields within the swarm, individual nuclei were first identified by watershed segmentation (SCF-MPI-CBG Fiji update site) of Gaussian and then median filtered (3D) raw images. The centroid of each labelled nuclei was used for cell tracking (TrackMate: simple LAP tracker, CSVImporter). Cell velocities were determined by the second-order central finite difference of cell positions. The mean cell velocity field across the swarm was calculated by averaging the velocity of each cell relative to the peak of the swarm (4μm (length) x 2μm (height) grid), averaged over a 15-minute period. The mean cell velocity field in the moving reference frame of the swarm was determined by subtracting the swarm velocity from the mean cell velocity field. The streamlines were determined by the Matlab streamline function. The Péclet number $P_e = Lu/D$ was calculated at each point in the travelling reference frame. Variable u (μm/min) is the mean cell speed (3D). Variable $L = A/S$ (μm) is the characteristic length, defined as the area of the swarm (viewed from the side), A, divided by the length of the curve that defines the swarm surface, S. Variable $D = \sigma^2 \Delta t/6$ (μm^2/min) is the diffusion coefficient at each grid point, where σ^2 is the variance of the instantaneous cell velocities (mean of the squared speeds relative to mean speed) at the grid point and $\Delta t = 4$s is the time between frames.

2. Mathematical Model

We derive a continuum mathematical model for the migration of *Dictyostelium* swarms based on the hypothesis that physical cell-cell interactions via the formation, attachment and retraction of pseudopods result in cell swarms having the following fluid-like material properties:

1) surface tension, κ, resulting from cell-cell adhesions (12);

2) viscosity, η, resulting from the transience of cell-cell adhesions (13);

3) bulk activity, ξ, generated by cells actively contracting as they move within the swarm (12, 14).

Here, we develop a minimal mathematical framework to model living droplets with a constant surface tension and viscosity, and a bulk activity that depends on a self-generated signal gradient. We aim to understand how these material properties contribute to the propagation and shedding of *Dictyostelium* populations during their directed migration in response to self-generated gradients in bacterial concentration.

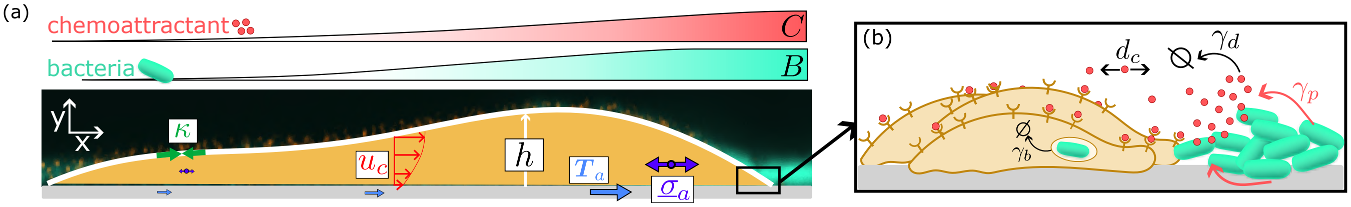

Fig. SM1. Schematics illustrating the model set-up and the mechanisms included. (a) The *Dictyostelium* swarm is represented as an active thin film of height $h(x,t)$. The flow of cells in the swarm is described by the flow field, $\boldsymbol{u}_c$, which is driven by an emergent surface tension (κ) and an emergent bulk contractile active stress ($\underline{\sigma}_a = \xi s^2(\boldsymbol{e}_x \otimes \boldsymbol{e}_x)$) and an emergent active traction force at the floor ($\boldsymbol{T}_a = \xi_T s \boldsymbol{e}_x$). Similar to classic lubrication theory, the velocity field is dominated by its horizontal component, which follows a Poiseuille-like profile (Section A.2). The scalar fields B and C indicate, respectively, the local concentration of the bacteria cells and the chemoattractant molecules produced by the bacteria (*e.g.*, folic acid). (b) *Dictyostelium* cells move up self-generated gradients in the concentration of chemoattractant molecules (red particles) that are produced by the bacterial cells. As *Dictyostelium* cells migrate, they feed on and deplete the bacterial population, thereby shaping chemoattractant gradients.

A. Full dynamical model of cell swarm migration: Pseudo-2D active thin-film model. We consider a (x,y) cross-section of the swarm and describe it as a thin 2D active polar fluid film (Fig. SM1) – a thin layer of fluid in which directed cell-cell interactions can drive fluid flows. As shown in Fig. SM1, the *Dictyostelium* swarm is described by the location of its free surface, *i.e.*, its height $y = h(x,t)$, with units of microns. The velocity field $\boldsymbol{u}_c$, with units of microns/minutes, describes the internal flow of *Dictyostelium* cells within the film. This represents a locally averaged velocity of *Dictyostelium* cells at each point in space and therefore captures the strength of any directed motion, which we assume to be driven by surface capillary forces (related to the effective surface tension κ), active traction forces between the cell and the surface and bulk active stresses, where the activity both at the surface and the bulk is regulated by the chemotactic signal, *i.e.*, the magnitude of chemoattractant gradients.The active driving forces are balanced by the frictional forces at the substrate that resist migration.

Because the swarm height (on the order of 10 μm) is small compared to its characteristic length (on the order of 100 μm), we adopt a lubrication approximation (15–17). For brevity, we present the reduced form of the governing equations obtained at the leading order in the lubrication limit since the derivation of the model via asymptotic methods follows standard approaches (16, 17).

A.1. Bacteria and chemoattractant concentration dynamics. We model the overall amount of bacterial cells at a given spatial location x via a continuous concentration field $B = B(x,t)$, with units cells/microns, which is consumed by cell groups at a rate proportional to its height (Fig. SM1b). Neglecting for simplicity any effect due to the advection of bacteria by the cell flow within the swarm, B evolves according to the following spatially-structured ODE:

$$\frac{\partial B}{\partial t} = -\gamma_b B h. \tag{S1}$$

In Eq. (S1) the term $\gamma_b h$, with units of 1/hour, is the rate of bacterial consumption within the cell swarm, where $\gamma_b > 0$ is a constant and $h(x,t)$ is the height of the swarm, as defined previously.

The chemoattractant concentration $C = C(x,t)$, with units of nanomolars, is modelled as a diffusible species with diffusion coefficient d_c. It decays at a constant rate, γ_d, while being produced at a rate γ_r by bacteria cells (Fig. SM1b). We assume the chemoattractant diffuses rapidly in the y-direction, in line with expected diffusive timescales in the lubrication limit. We neglect any advection of the chemoattractant by *Dictyostelium* cell movement. The time evolution of C is dictated by the following reaction-diffusion partial differential equation:

$$\frac{\partial C}{\partial t} = d_c \frac{\partial^2 C}{\partial x^2} - \gamma_d C + \gamma_p B. \tag{S2}$$

The direction of droplet movement is dependent on the initial positioning of the bacterial lawn, *i.e.*, the initial condition for B, which introduces some pre-patterning that breaks the left-right symmetry in the system. We discuss this further in Section B.

A.2.* Dictyostelium *cell movement. We assume that the total stress within the swarm

$$\underline{\sigma} = -p\underline{\mathrm{I}} + \eta[\nabla \boldsymbol{u}_c + (\nabla \boldsymbol{u}_c)^T] + \xi s^2 \left(\boldsymbol{e}_x \otimes \boldsymbol{e}_x - \frac{1}{2}\underline{\mathrm{I}}\right) \tag{S3}$$

is the sum of three contributions: the pressure p, a viscous stress with shear viscosity η, in units of Pascals·minutes, and an active stress with constant activity $\xi > 0$, in units of Pascals. In defining the active component of the stress (final bracketed term on the right-hand side of Eq. (S3)), we have assumed any nematic order (or cell polarity) to be directed along the vector $\boldsymbol{e}_x$, *i.e.* in the direction of the chemoattract gradient. The strength of the nematic order parameter $s^2 \in [0,1]$ depends on the magnitude of the chemoattractant gradient and is discussed in detail below. In the lubrication limit, the movement of cells in the y-direction is negligible and the velocity field $\boldsymbol{u}_c \approx u_c \boldsymbol{e}_x$. In this regime, the stress in the fluid is dominated by the pressure p, which is obtained by imposing force balance at the air-swarm interface along the direction normal to the free-surface (15):

$$p = -\kappa \left[\frac{\partial^2 h}{\partial x^2} - \Psi(h)\right] - \frac{\xi s^2}{2}. \tag{S4}$$

The first two terms on the right-hand side of Eq. (S4) are common in the modeling of passive droplets and capture respectively the capillary forces, which result from an effective constant surface tension κ, in units of Netwons per microns, and a disjoining pressure Ψ resulting from the interaction of cells with the surface, which gives rise to an emergent macroscopic contact angle (θ_e). We here use a standard form for Ψ (15):

$$\Psi(h) = \frac{3h_\delta^2 \tan^2\theta_e}{(h+h_\delta)^3}\left(1 - \frac{h_\delta}{h+h_\delta}\right). \qquad \text{[S5]}$$

In Eq. (S5) θ_e is the equilibrium contact angle for the film and h_δ is a correction introduced to allow for dewetting without introducing a pre-wetting layer. Here, we take h_δ to be half the size of a *Dictyostelium* cell ($h_\delta = 5\mu$m) so that Ψ describes effective attractive interactions between the layer of cells in contact with the floor and the floor itself. These forces allow for film rupture when cell layers are extremely thin (18) ($h < h_\delta$), but otherwise have a negligible effect. For a static film, Eq. (S5) fixes the contact angle with floor to be θ_e; however, for a moving thin film, Eq. (S5) dynamically regulates the contact angle.

We hypothesise that cell-floor interactions allow cells to move along the floor by exerting a traction force, while experiencing an effective friction. The simplest mathematical model that accounts for both of these effects is a modified Navier slip condition at the floor (16, 19):

$$u_c - \xi_T s - \ell_s \frac{\partial u_c}{\partial y} = 0, \quad y = 0, \qquad \text{[S6]}$$

where $\ell_s > 0$, with units of microns, represents the slip length, which is inversely proportional to the cell-floor friction. The function ξ_T instead captures the maximum directed velocity of the cell at the floor mediated by the ability of cells to exert traction forces on the substrate and $s \in [-1, 1]$ captures the magnitude of cell polarisation in the direction of the chemical signal. With these assumptions, we find that u_c follows a leading-order parabolic profile:

$$u_c(x, y, t) = \frac{F(x,t)}{\eta}\left[h(x,t)\,(\ell_s + y) - \frac{y^2}{2}\right] + \xi_T s. \qquad \text{[S7a]}$$

The function F describes the net force density that drives cell movement; this consists of three terms:

$$F(x,t) = \kappa\frac{\partial^3 h}{\partial x^3} - \kappa\frac{\partial \Psi(h)}{\partial x} + \xi\frac{\partial s^2}{\partial x}. \qquad \text{[S7b]}$$

The profile of the horizontal velocity in Eq. (S7) is obtained by integrating the x-component of the momentum conservation equation ($\eta\partial_{yy}u_c = \partial_x\left[p - s^2\xi/2\right]$), balancing surface forces in the direction tangential to the air-swarm free-surface ($\partial_y u_c|_{y=h(x,t)} = 0$), and imposing the floor condition (S6) within the lubrication limit.

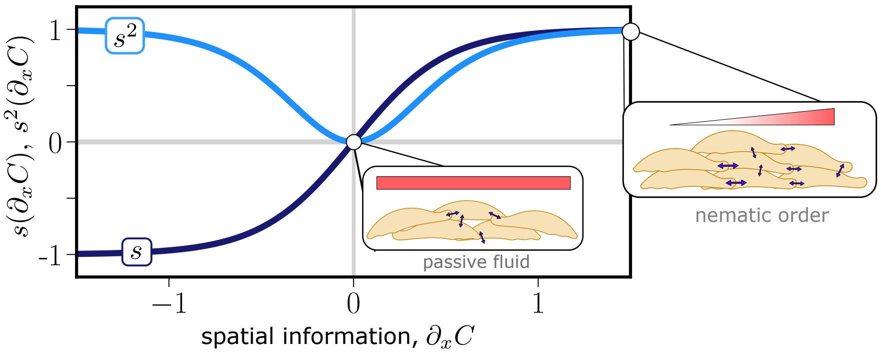

Fig. SM2. Modulation of cell polarisation, s, and polar alignment, s^2, by chemoattractant gradients (Eq. (S8)). If chemoattractant gradients are small, cells lack information regarding the location of the bacteria and their movement is random ($s \approx 0$). When chemoattractant gradients are larger, the bias of cell movement is in the direction of the chemoattractant gradients and the alignment function increases linearly until it saturates at $\|s(x)\| \approx 1$, which corresponds to perfect bias of cell movement in the direction of increasing bacteria concentration.

Practically, *Dictyostelium* cells achieve directed migration by biasing the distribution of pseudopods (14). In the absence of any directional information, the distribution of pseudopods, and therefore cell movement, is random. Chemotaxis is thought to bias random cell motility, by favouring retention of the pseudopod that experiences the higher attractant concentration. The stronger the chemotactic signal, the stronger the bias. In our model, chemotaxis bias is introduced by cell alignment function $s \in [-1, 1]$ to the chemotactic signal. When $s \approx 0$, cell movement is random and the collective behaves effectively as a passive fluid. When $|s| = 1$, cell movement is strongly biased and the strong alignment in the direction of cell contractility results in maximal active stresses and traction at the floow (20). For simplicity, we assume a sigmoidal dependence of the local alignment parameter on the chemotactic signal, here generally denoted by ω:

$$s_\alpha(\omega) = \tanh(\alpha\omega), \qquad \text{[S8]}$$

where $\alpha > 0$ is a positive constant that controls the width of the interval in which the alignment s increases linearly and its gradient. Eq. (S8) accounts for the loss of positional information when the response function is small, and for the saturation on

the local strength of alignment when all cells are completely polarised. In practice, *Dictyostelium* cells will sense gradients in receptor occupancy, which can yield random cell movement at high chemoattractant concentrations as a result of receptor saturations (21, 22). Here we assume that the chemoattractant concentration remains below the saturation threshold of the binding sites ($\approx 20\,\mathrm{nM}$ (22)), so that cells respond to gradients in the chemoattractant, and set $\omega = \partial_x C$ (Fig. SM2).

A.3. Dictyostelium *swarm dynamics.* The time-evolution of the height function h is determined by imposing mass balance. Given that the density of cells within the droplet is assumed to be constant, we find that:

$$\frac{\partial h}{\partial t} + \frac{\partial Q}{\partial x} = \frac{rB^2}{m_B^2 + B^2} h, \qquad \text{[S9a]}$$

where the flow rate Q is obtained by integrating the horizontal cell velocity in Eq. (S7a) over y:

$$Q(x,t) = \int_0^h u_c(x,y,t)\,dy = \frac{F(x,t)}{\eta} h^2(x,t) \left[\ell_s + \frac{h(x,t)}{3}\right] + \xi_T s_\alpha h(x,t), \qquad \text{[S9b]}$$

$$F(x,t) = \kappa \frac{\partial^3 h}{\partial x^3} - \kappa \frac{\partial \Psi(h)}{\partial x} + \xi \frac{\partial s_\alpha^2}{\partial x}. \qquad \text{[S9c]}$$

In Eq. (S9a), the rate of cell proliferation is taken to be a non-linear function of the concentration of bacteria. Cells proliferate at their maximum rate ($r > 0$, with units 1/minutes) as long as there is enough food, while they arrest when starved. Here, $m_B > 0$, in units of cells/microns, indicates the concentration of bacteria below which cell proliferation is arrested.

Looking at Eq. (S9), we find that swarm migration is driven by two active forces: active traction that results from cells pulling on the surface to propel and gradients in the active stresses that arise at the population level through cell-cell interactions at the microscopic level. The former results in an effective advection term, $\xi_T s_\alpha h$, which is non-linearly dependent on the chemoattractant gradients via the alignment function s_α (see Eq. (S8)). For relatively small chemoattractant gradients, $s_\alpha(\partial_x C) \approx \partial_x C$ and the traction term reduces to the same term included in the standard Keller-Segel model (23). While this term plays a dominant role at low cell concentrations, the emergent swarm dynamics and flow profiles observed experimentally suggest that this term is negligible (more details in Section D). Hence we will consider from now on $\xi_T = 0$. The comparison with experimental data in Section D suggests instead that the primary mechanism responsible for the migration of *Dictyostelium* cell groups is the gradients in the active stress. The constant ξ in Eq. (S9c) is the activity parameter that measures the typical size of the active stresses. Generally, ξ can be of either sign. Using the same sign convention as in (17), here we take $\xi > 0$, under the assumption that *Dictyostelium* cell activity generates contractile forces in the preferred direction of motion through pseudopods that temporarily attach to other neighbouring cells (14, 24).

A.4. Non-dimensional model. We non-dimensionalise the governing equations using the following scalings:

$$\begin{gathered} x = L\hat{x}, \quad y = \tan\theta_e L\hat{y}, \quad t = \frac{L}{U}\hat{t} \quad u_c = U\hat{u}_c, \\ h = \tan\theta_e L\hat{h}, \quad B = B_\infty \hat{B}, \quad C = \frac{\gamma_p B_\infty}{\gamma_d}\hat{C}, \end{gathered} \qquad \text{[S10]}$$

where L is the characteristic length of the swarm, B_∞ is the characteristic concentration of bacteria, U is the characteristic velocity of the cells within the swarm and θ_e is the equilibrium contact angle. By substituting Eqs. (S10) into Eqs. (S1)-(S2) and (S7)-(S9), we obtain the following non-dimensional system of coupled partial differential equations for $\hat{B}$, $\hat{C}$ and $\hat{h}$:

$$\text{Bacteria consumption} \quad : \quad \frac{\partial \hat{B}}{\partial \hat{t}} = -E\hat{B}\hat{h}, \qquad \text{[S11a]}$$

$$\text{Diffusion and production of chemoattractant} \quad : \quad \frac{\partial \hat{C}}{\partial \hat{t}} = D_c \frac{\partial^2 \hat{C}}{\partial \hat{x}^2} - \Gamma_d \left(\hat{C} - \hat{B}\right), \qquad \text{[S11b]}$$

$$\text{Dynamics of the thin-film height} \quad : \quad \frac{\partial \hat{h}}{\partial \hat{t}} = -\frac{\partial}{\partial \hat{x}}\left(M(\hat{h})\frac{\partial \pi}{\partial \hat{x}}\right) + \frac{R\hat{B}^2}{\hat{m}_B^2 + \hat{B}^2}\hat{h}, \qquad \text{[S11c]}$$

where the mobility M and the pressure π are defined as:

$$M(\hat{h}) = \frac{1}{Ca_\kappa}\hat{h}^2\left(\frac{\hat{h}}{3} + L_s\right), \qquad \text{[S11d]}$$

$$\pi = \frac{\partial^2 \hat{h}}{\partial \hat{x}^2} - \frac{3H_\delta^2}{(\hat{h} + H_\delta)^3}\left(1 - \frac{H_\delta}{\hat{h} + H_\delta}\right) + Ca_\xi \hat{S}_{\hat{\alpha}}\left(\frac{\partial \hat{C}}{\partial \hat{x}}\right), \qquad \text{[S11e]}$$

In Eq. (S11e), the pressure accounts for three contributions: 1) the capillary pressure, which accounts for surface tension; 2) the disjoining pressure, which mediates droplet contact angles and splitting; and, 3) the active pressure, which accounts for chemotactic cell interactions – this is mediated by the rescaled order parameter $\hat{S}(\cdot) = 3\sqrt{3}/(4\hat{\alpha})s_{\hat{\alpha}}^2(\cdot)$, defined in Eq. (S8), with

rescaled hyper-parameters $\hat{\alpha}$. In the non-dimensional form of the model, the evolution of the swarm height $\hat{h}$ is determined by seven non-dimensional parameters:

$$L_s = \frac{\ell_s}{L\tan\theta_e}, \quad Ca_\kappa = \frac{U\eta}{\kappa(\tan\theta_e)^3}, \quad Ca_\xi = \frac{4\xi L\hat{\alpha}}{3\sqrt{3}\tan\theta_e\kappa}, \\ R = \frac{rL}{U}, \quad H_\delta = \frac{h_\delta}{L\tan\theta_e}, \quad \hat{\alpha} = \frac{\alpha\gamma_p B_\infty}{L\gamma_d}, \tag{S12a}$$

and three non-dimensional parameters determine the evolution of $\hat{B}$ and $\hat{C}$:

$$D_c = \frac{d_c}{UL}, \quad E = \frac{\gamma_b L^2\tan\theta_e}{U}, \quad \Gamma_d = \frac{\gamma_d L}{U}. \tag{S12b}$$

The model is closed by imposing boundary and initial conditions. These are discussed in Section B.

B. Numerical simulation of the shedding dynamics. To simulate the experimental conditions, we solve the dimensionless governing equations (Eqs. (S11)) on a large, finite domain, $\hat{x} \in [0, X]$, where $X \gg 1$. Non-dimensional parameter values are discussed in Section D and listed in Table SM2.

B.1. Boundary conditions. We impose no flux boundary conditions for both $\hat{C}$ and $\hat{h}$ at either side of the domain:

$$\partial_{\hat{x}}\hat{C} = 0, \quad \hat{x} \in \{0, X\}, \tag{S13a}$$

$$M(\hat{h})\frac{\partial\pi}{\partial\hat{x}} = 0, \quad \hat{x} \in \{0, X\}, \quad t > 0. \tag{S13b}$$

Since the equation describing the evolution of $\hat{h}$ is fourth-order in x, an additional pair of boundary conditions for $\hat{h}$ is needed. We apply the natural boundary conditions:

$$\partial_{\hat{x}}\hat{h} = 0, \quad \hat{x} \in \{0, X\}, \tag{S13c}$$

which correspond to vanishing contact angles at the boundaries, which are appropriate since we guarantee the droplet remains far enough from the domain boundaries.

B.2. Initial conditions. We set the initial swarm to be a small symmetric Gaussian droplet of width $\sigma_h = \sqrt{0.1}$ centred at location $\hat{x}_0$ sufficiently far from the domain boundary (see function $\hat{h}_0$ in Fig. SM3). We replicate the bacterial tracks in the 1D experiments (Fig. S7) by considering an initially monotonically increasing bacterial profile (Fig. SM3) which saturates away from the front of the swarm, $\hat{B}_0(x) \approx 1$ for $\hat{x} \gg \hat{x}_0$, and decays to zero behind the swarm, $\hat{B}_0(\hat{x}) \approx 0$ as $\hat{x} \ll \hat{x}_0$. Without loss of generality, we equate the characteristic bacterial concentration B_∞ used to scale the bacterial concentration (Eq. (S10)), to the far-field bacterial concentration ahead of the swarm. We further assume that $\hat{C}_0 = \hat{B}_0$, so that it satisfies the equilibrium of the reaction term in Eq. (S11b).

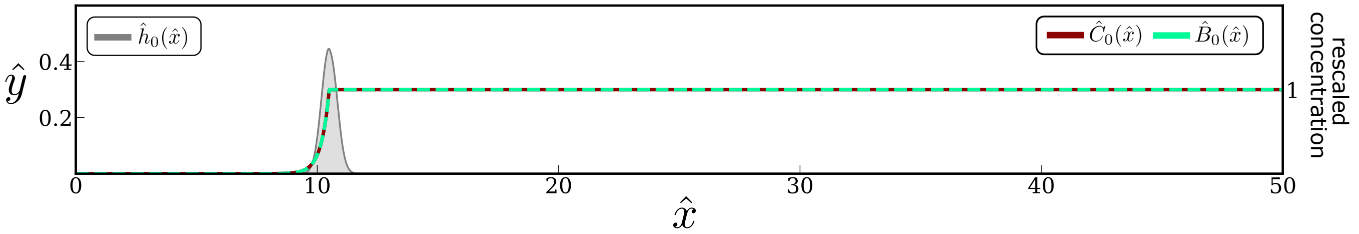

Fig. SM3. Plot showing the initial conditions used in the simulations of the swarm dynamics.

B.3. Numerical scheme. The full model requires solving three coupled non-linear partial differential equations for $\hat{h}$, $\hat{B}$ and $\hat{C}$, Eqs. (S11)-(S13). We adopt a semi-implicit time-discretization to decouple the three equations and advance them over time (time-step size $\delta t = 0.001$). Specifically, given the approximate solution at time $t = \hat{t}_j$, $\left(h^j(\hat{x}) = \hat{h}(\hat{t}_j, \hat{x}),\, B^j(\hat{x}) = \hat{B}(\hat{t}_j, \hat{x})\right.$ and $\left.C^j(\hat{x}) = \hat{C}(t_j, \hat{x})\right)$, we proceed as follows:

1. We compute h^{j+1} using Eq. (S11c) and setting $\hat{C} = C^j$ and $\hat{B} = B^j$.
2. We compute B^{j+1} using Eq. (S11a) and setting $\hat{h} = h^{j+1}$.
3. We compute C^{j+1} using Eq. (S11b) and setting $\hat{B} = B^{j+1}$.

We use first-order Lagrangian finite elements to discretise the governing equations in space and divide the domain into equal intervals of size $\delta x = 0.02$. We adopt a semi-implicit time-stepping scheme to deal with the non-linar motility function M in Eq. (S11c), *i.e.*, we evaluate M explicitly in time $M(\hat{h}) = M(h^j)$. The code is implemented in `python`, based on the `FEniCS` package for finite element methods (25). The code is freely available at https://github.com/giuliacelora/Dictyostelium-Swarm-Migration.

For numerical convenience, we also introduce a small level of artificial diffusion in solving the spatially-structured ODE for $\hat{B}$, with diffusion coefficient in non-dimensional units $d_b = 10^{-4}$; this is coupled to no-flux boundary conditions on $\hat{B}$ at both ends of the simulation domain.

C. Travelling-wave model of swarm migration: Pseudo-2D active droplet. As shown in the main text, we can identify parameter regimes for which the proposed thin-film model can replicate the two-phase (travelling and shedding) swarm migration dynamics observed experimentally. The shedding of the swarm is initiated by a rapid elongation of the swarm that transitions from being a compact droplet to a multi-peaked asymmetric droplet. We are interested in understanding which mechanisms dictate this transition. We hypothesise that the elongation is driven by an imbalance between the capillary and active forces that drive the flow of cells within the droplet (away from the contact angles where the disjoining pressure becomes relevant).

To test this hypothesis, we consider a simplified version of the model for a single, self-confined droplet whose mass increases quasi-statically; this model captures all of the key features of the thin-film model presented in Section A, but it is more amenable to mathematical analysis. Since we are interested in capturing solutions in which the cells are confined within a single droplet, we formulate the model into a free boundary problem, where we explicitly follow the location of the contact line, $\hat{x}_{R,F}(t)$, corresponding to the rear and front boundaries of the swarm. For simplicity, we pose the governing equations in non-dimensional form. Under the above assumptions, Eqs. (S11) reduce to:

$$\frac{\partial \hat{B}}{\partial \hat{t}} = -E\hat{B}\hat{h}, \qquad \hat{x} \in \mathbb{R}, \qquad \text{[S14a]}$$

$$\frac{\partial \hat{C}}{\partial \hat{t}} = D_c \frac{\partial^2 \hat{C}}{\partial \hat{x}^2} - \Gamma_d \left(\hat{C} - \hat{B}\right), \qquad \hat{x} \in \mathbb{R}, \qquad \text{[S14b]}$$

$$\frac{\partial \hat{h}}{\partial \hat{t}} = -\frac{1}{Ca_\kappa}\frac{\partial}{\partial \hat{x}}\left(\hat{h}^2\left(\frac{\hat{h}}{3} + L_s\right)\frac{\partial \pi}{\partial \hat{x}}\right) + \frac{R\hat{B}^2}{\hat{m}_B^2 + \hat{B}^2}\hat{h}, \qquad \hat{x} \in (\hat{x}_R(t), \hat{x}_F(t)), \qquad \text{[S14c]}$$

$$\pi = \frac{\partial^2 \hat{h}}{\partial \hat{x}^2} + Ca_\xi \hat{S}_{\hat{\alpha}}\left(\frac{\partial \hat{C}}{\partial \hat{x}}\right), \qquad \text{[S14d]}$$

where the swarm boundaries are implicitly defined, imposing that the droplet height first takes the value zero at these points:

$$\hat{h}(\hat{x}_R(\hat{t}), t) = \hat{h}(\hat{x}_F(\hat{t}), t) = 0, \qquad \text{[S14e]}$$

and the boundary conditions for the chemoattractant fields are:

$$\lim_{\hat{x}\to\infty} \hat{C}(\hat{x}, \hat{t}) = 1, \quad \lim_{\hat{x}\to-\infty} \frac{\partial \hat{C}}{\partial \hat{x}}(\hat{x}, \hat{t}) = 0. \qquad \text{[S14f]}$$

The dynamics of the floor-droplet contact points are determined by imposing conservation of mass at the moving contact lines:

$$\left.\frac{\partial \hat{h}}{\partial \hat{t}}\right|_{\hat{x}=\hat{x}_i} + \hat{x}_i'(\hat{t}) \left.\frac{\partial \hat{h}}{\partial \hat{x}}\right|_{\hat{x}=\hat{x}_i} = 0, \quad i \in \{R, F\}. \qquad \text{[S14g]}$$

We note that we have dropped the term associated with disjoining pressure in the definition of π in Eq. (S14d). This is because, in modeling a droplet, the contact angles are strongly imposed at the contact lines. Here, we use a dynamic contact angle model derived in (26) to capture the recession and advancement motion of the contact lines (Fig. SM4) for the moving cell group observed in the dynamic simulations. In this framework, contact angles are determined by the following boundary conditions

$$Ca_\theta \hat{x}_i'(\hat{t}) = \left.\left(\left(\frac{\partial \hat{h}}{\partial \hat{x}}\right)^2 - 1\right)\right|_{x=\hat{x}_i} n_i, \quad i \in \{R, F\}, \qquad \text{[S14h]}$$

where $Ca_\theta = 2U\eta_\theta/(\kappa \tan\theta_e^2)$ is the contact angle capillary number measuring the ratio between the energy dissipation at the contact lines – characterised by the friction coefficient η_θ – and n_i indicates the outer normal at the contact lines, *i.e.*, $n_R = -1$ and $n_F = 1$. Eq. (S14) is derived assuming energy is dissipated at the contact line of a moving droplet. In the case of a static droplet, the right-hand side of Eq. (S14) vanishes and we recover the equilibrium contact angle condition ($\partial_{\hat{x}}\hat{h} = 1 \Rightarrow \partial_x h = \tan\theta_e$). We note that the use of Eq. (S14h) is phenomenological rather than mathematically equivalent to the modulation of the contact lines in the full dynamic simulations. The model is closed by imposing appropriate initial conditions (Section B.2).

C.1. Travelling-wave analysis. We exploit the separation between the proliferation ($\sim$ hrs) and hydrodynamic time scales ($L/U \sim$ min) and assume that the free surface can rapidly adjust to proliferation-driven changes in the swarm volume. Hence, we adopt a quasi-steady approximation: upon small changes in the droplet mass due to proliferation, the surface $\hat{h}$, as well as the fields $\hat{B}$ and $\hat{C}$, relax to a profile that is steady in an appropriate travelling frame. The shape of the profile and the velocity of the travelling frame have to be determined as part of the solution and depends on the volume of the droplet, ν_{TW}. Effectively, we therefore model the migrating cell group as a self-contained active droplet with a quasi-constant volume. We compute travelling-wave solutions by introducing the moving reference frame

$$\hat{\varphi} = \hat{x} - U_{\text{TW}}\hat{t}, \tag{S15}$$

where U_{TW} is the unknown velocity of the travelling-wave that we have to determine as part of our solution. We then substitute the travelling-wave ansatz

$$\hat{h}(\hat{x},\hat{t}) = h_{\text{TW}}(\hat{\varphi}), \quad \hat{B}(\hat{x},\hat{t}) = B_{\text{TW}}(\hat{\varphi}), \quad \hat{C}(\hat{x},\hat{t}) = C_{\text{TW}}(\hat{\varphi}), \tag{S16}$$

into Eq. (S14) to obtain:

$$U_{\text{TW}} h_{\text{TW}} = \frac{1}{Ca_\kappa} h_{\text{TW}}^2 \left[L_s + \frac{h_{\text{TW}}}{3}\right] \partial_{\hat{\varphi}} \pi_{\text{TW}}, \quad \hat{\varphi} \in [0, L_{\text{TW}}], \tag{S17a}$$

$$\pi_{\text{TW}} = \partial_{\hat{\varphi}\hat{\varphi}} h_{\text{TW}} + Ca_\xi \hat{S}_{\hat{\alpha}}(\partial_{\hat{\varphi}} C_{\text{TW}}), \tag{S17b}$$

with boundary conditions:

$$h_{\text{TW}}(0) = h_{\text{TW}}(L_{\text{TW}}) = 0, \tag{S17c}$$

$$\partial_{\hat{\varphi}} h_{\text{TW}}(0) = \sqrt{1 - Ca_\theta U_{\text{TW}}}, \tag{S17d}$$

$$\partial_{\hat{\varphi}} h_{\text{TW}}(L_{\text{TW}}) = -\sqrt{1 + Ca_\theta U_{\text{TW}}}. \tag{S17e}$$

The length L_{TW} is an unknown set by the constraining the volume of the droplet:

$$\int_0^{L_{\text{TW}}} h_{\text{TW}}(\hat{\varphi}) d\hat{\varphi} = \nu_{\text{TW}}. \tag{S17f}$$

Eq. (S17) appears similar to the travelling wave problem that describes the movement of passive droplets under gravity (26). However, there are key physical and mathematical difference. Physically, active droplets move without the influence of an external field since energy is generated at the micro-scale. Mathematically, the active forcing in our model is spatially-heterogeneous and self-regulated –*i.e.*, it non-linearly depends on the travelling-wave solution via its coupling with $\partial_{\hat{\varphi}} C_{\text{TW}}$. The bacterial and chemoattract concentration profiles are determined by the following system:

$$U_{\text{TW}} \partial_{\hat{\varphi}} B_{\text{TW}} - E h_{\text{TW}} B_{\text{TW}} = 0, \tag{S18a}$$

$$D_c \partial_{\hat{\varphi}\hat{\varphi}} C_{\text{TW}} + U_{\text{TW}} \partial_{\hat{\varphi}} C_{\text{TW}} + \Gamma_d (B_{\text{TW}} - C_{\text{TW}}) = 0, \tag{S18b}$$

with boundary conditions:

$$\lim_{\hat{\varphi}\to\infty} B_{\text{TW}}(\hat{\varphi}) = 1, \quad \lim_{\hat{\varphi}\to-\infty} D_c \partial_{\hat{\varphi}} C_{\text{TW}} = 0, \quad \lim_{\hat{\varphi}\to\infty} C_{\text{TW}}(\hat{\varphi}) = 1. \tag{S18c}$$

Eq. (S18) are derived substituing Eq. (S15) into Eqs. (S14a)-(S14b) and Eq. (S14f). The far-field condition on B_{TW} comes from the choice of initial conditions, which replicates the experimental 1D track experiment (Section B).

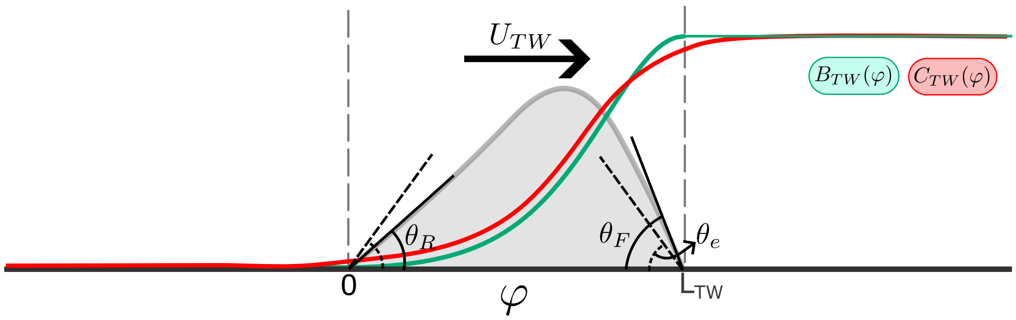

Fig. SM4. Schematic illustrating how we construct the travelling wave solutions for self-contained droplet without proliferation moving at speed U_{TW}. Eq. (S14h) is such that the front contact line will be advancing ($\theta_F > \theta_e$) while the rear contact line is receding ($\theta_F < \theta_e$).

We solve Eqs. (S18) explicitly for B_{TW} and C_{TW} as a function of h_{TW} (Fig. SM4):

$$B_{\mathrm{TW}}(\hat{\varphi}) = \begin{cases} 1, & \hat{\varphi} > L_{\mathrm{TW}}, \\ \exp\left[-\frac{E}{U_{\mathrm{TW}}}\int_{\hat{\varphi}}^{L_{\mathrm{TW}}} h_{\mathrm{TW}}(\xi)d\xi\right], & 0 < \hat{\varphi} \leq L_{\mathrm{TW}}\ , \\ \exp\left[-\frac{E\nu_{\mathrm{TW}}}{U_{\mathrm{TW}}}\right], & \hat{\varphi} \leq 0. \end{cases} \tag{S19a}$$

$$C_{\mathrm{TW}}(\hat{\varphi}) = \begin{cases} 1 + \left[C(L_{\mathrm{TW}}) - 1\right] e^{\lambda_-(\hat{\varphi} - L_{\mathrm{TW}})}, & \hat{\varphi} > L_{\mathrm{TW}}, \\ D_c\left[\lambda_- G(\hat{\varphi}; L_{\mathrm{TW}}) - e^{-\frac{E\nu_{\mathrm{TW}}}{U_{\mathrm{TW}}}}\lambda_+ G(\hat{\varphi}; 0)\right] \\ \quad -\Gamma_d \displaystyle\int_0^{L_{\mathrm{TW}}} G(\hat{\varphi}; w) B_{\mathrm{TW}}(w) dw, & 0 \leq \hat{\varphi} \leq L_{\mathrm{TW}}, \\ B_{\mathrm{TW}}(0) + \left[C_{\mathrm{TW}}(0) - B_{\mathrm{TW}}(0)\right] e^{\lambda_+ \hat{\varphi}}, & \hat{\varphi} < 0, \end{cases} \tag{S19b}$$

where $\lambda_\pm$ are defined as:

$$\lambda_\pm = \frac{-U_{\mathrm{TW}} \pm \sqrt{U_{\mathrm{TW}}^2 + 4D_c\Gamma_d}}{2D_c}. \tag{S19c}$$

and $G(\hat{\varphi}; \xi)$ is the Green's function associated with Eq. (S18b):

$$G(\hat{\varphi}; w) = \begin{cases} \dfrac{1}{D_c}\dfrac{e^{\lambda_+(\hat{\varphi} - w)}}{\lambda_- - \lambda_+}, & 0 \leq \hat{\varphi} < w, \\ \dfrac{1}{D_c}\dfrac{e^{\lambda_-(\hat{\varphi} - w)}}{\lambda_- - \lambda_+}, & w < \hat{\varphi} \leq L_{\mathrm{TW}}, \end{cases} \tag{S19d}$$

Given the semi-explicit form of B_{TW} and C_{TW}, Eqs. (S19), for any given value of the swarm volume ν_{TW}, we solve for h_{TW}, L_{TW} and U_{TW} numerically using the Newton-Krylov method (27). We discretize Eq. (S17) using a finite volume discretisation (with a total of $N = 100$ cells). We use numerical continuation to solve the boundary value problem as a function of the swarm volume, ν_{TW}. The numerical continuation is implemented in `Julia` using the `BifurcationKit.jl` for automatic bifurcation analysis (27). The `Julia` code is freely available at https://github.com/giuliacelora/Dictyostelium-Swarm-Migration.

Flow profile in the travelling wave regime We here discuss how we obtain the model predictions on cell velocity profiles shown in Fig. 5 of the main text. Substituting the travelling-wave conditions (S17a)-(S17b) into Eq. (S7a) and incorporating the appropriate scaling in Eq. (S10)), we find that the dimensionless horizontal flow field for travelling-wave solutions is:

$$u_c^{TW}(\varphi, y) = \frac{u_{\mathrm{TW}}}{h_{\mathrm{TW}}(\varphi)\left(\ell_s + \frac{h_{\mathrm{TW}}(\varphi)}{3}\right)}\left[h_{\mathrm{TW}}(\varphi)\left(\ell_s + y\right) - \frac{y^2}{2}\right], \quad \varphi \in [0, \ell_{\mathrm{TW}}], \tag{S20}$$

where u_{TW}, $\ell_{\mathrm{TW}} = L_{\mathrm{TW}}$ and $\varphi = L\hat{\varphi}$ are the dimensional travelling-wave velocity, width and reference frame, respectively. Eq. (S20) is used to generate the plot in Figs. 5A and 5F. To generate the full flow field in Fig. 5B, we extend our lubrication analysis to approximate the leading-order expansion of the vertical component of the cell velocity v_c^{TW}. While this is negligible when evaluating the swarm migration, it is key to obtain the full circulatory (vortex-like) profile of cells within the swarm. In the travelling-wave analysis, proliferation is neglected and the volume of the droplet is considered to be constant. Hence the flow $\boldsymbol{u}_c^{TW}$ is incompressible, where $\boldsymbol{u}_c^{TW}(\varphi, y) = u_c^{TW}(\varphi, y)\boldsymbol{e}_x + v_c^{TW}(\varphi, y)\boldsymbol{e}_y$ (both in the Eulerian and travelling-wave reference frames). We obtain v_c^{TW} by integrating the incompressibility condition and imposing a no-penetration condition at the floor ($v_c^{TW}(\varphi, 0) = 0$):

$$v_c^{TW}(\varphi, y) = \frac{3u_{\mathrm{TW}}h_{\mathrm{TW}}(\varphi)}{h_{\mathrm{TW}}^2(3\ell_s + h_{\mathrm{TW}}(\varphi))^2}\left[y^3\left(\frac{\ell_s}{2} + \frac{h_{\mathrm{TW}}(\varphi)}{3}\right) - h_{\mathrm{TW}}^2(\varphi)\left(y\,\ell_s + \frac{y^2}{2}\right)\right]. \tag{S21}$$

Eqs. (S20)-(S21) are used to generate Fig. 5B, where the travelling wave solution is chosen such that $\ell_{\mathrm{TW}} = 460\mu m$, which is the length of the droplet after shedding in the dynamical simulations. We note that there are two compact travelling-wave solutions (*i.e.*, both solutions belonging to the branch of the yellow curve in Fig. SM7a below the critical point) that satisfy this condition due to the non-monotonic dependence of the travelling wave velocity and the volume ν_{TW}. We select the travelling wave solution with the largest volume. This does not affect the qualitative nature of the velocity profile.

D. Parameter values. The dimensional values of the physical parameters in the thin-film model of cell swarm migration are listed in Table SM1. We estimate most of the parameters from either the literature or experimental data (see Table SM1). The α parameter that controls the dependence of the alignment on the local chemoattractant gradients (see Eq. (S8)), and the threshold concentration of bacteria for Dictyostelium cell growth m_B (see Eq. (S9a)) are chosen arbitrarily within the range of physically realistic values. Provided that they satisfy the physical constraints indicated in Table SM1, their precise values only have a minor influence on the numerical simulations and the inferred values of the swarm emergent material properties, *i.e.*, κ/ξ and κ/η, that best capture the experimental dynamics.

The corresponding values of the non-dimensional parameters (see Eqs. (S12)) adopted for the simulations of the thin-film model (Eqs. (S11)) and the travelling-wave analysis (Eqs. (S17) and (S19)) are given in Table SM2. While the thin-film and droplet models of cell swarm migration presented in Sections A and C share most of the physical parameters, there is a difference in how the contact angle between the cell swarm and the surface is imposed. In the thin-film model, this is controlled by the disjoining pressure (Eq. (S5)). In the droplet model, the contact angles are regulated by the boundary condition (S14h), which depends on a single non-dimensional parameter: the contact angle capillary number Ca_θ; the value of Ca_θ in Table SM2 is chosen to match the maximum value of the travelling wave velocity U_{TW} (see Fig. 3G main text) with the maximum value of the front velocity observed in the dynamical simulations (see Fig. 3E main text).

Parameter	Meaning	Value	Justification
L	Characteristic swarm width	100 [μm]	(exp) – Fig. 2 main text
U	Characteristic Dictyostelium cell speed	12.5 [μm/min]	(exp) – Fig. 2 main text
B_∞	Concentration of bacteria in the lawn	1 [a.u.]	w.l.o.g.
C_∞	Concentration of chemoattractant in the lawn ($C_\infty = \gamma_p B_\infty/\gamma_d$)	100 [a.u.]	w.l.o.g.
θ_{eq}	Equilibrium contact angle for shedded groups	33 degree	(exp) – Fig. S3
κ/η	visco-capillary speed	262.4 [μm/min]	(exp) – Fig. SM6
κ/ξ	Ratio between emergent surface tension and emergent activity of the cell group	41.2 [μm]	(exp) – Fig. SM6
ξ_T	active surface-traction speed	0.0 [μm]	(exp) – Fig. SM5
r	replication rate of Dictyostelium cells growing on bacteria (doubling time 4 hr)	0.15 [1/hr]	(28)
ℓ_s	Slip length characterising friction between cell and the surface	5.2 [μm]	(exp) – Fig. SM5
γ_b	Consumption rate of bacteria per unit swarm height	0.0387 [1/(μm hr)]	(exp) – Fig. SM5
d_c	Chemoattractant diffusion coefficient	12×10^3 [(μm)^2/min]	(22)
γ_d	Decay rate of the chemoattractant	1.2 [1/min]	decay length $L_c = \sqrt{d_c/\gamma_d}$ set to be 100μm, in line with previous theoretical studies (29)
h_δ	Length scale at which attractive cell-floor interactions dominate	5 [μm]	taken to be half the size of a cell
α	Steepness of the alignment curve $s_\alpha(\partial_x c)$ (see Eq. (S8))	2 [a.u./μm]	taken to be 100 times smaller than β
m_B	Threshold for arrest of cell proliferation	0.05 [a.u.]	taken to be small compared to B_∞

Table SM1. List of the dimensional physical parameters in Eqs. (S1)-(S9) and the values used in the simulation presented in the main text. The parameters labelled with (exp) are estimated from measured experimental data (see Section D for details). The highlighted parameter groupings characterise the emergent properties of the cell swarm that cannot be measured directly but are inferred to match the emergent shedding dynamics of model prediction to 1D track experiments (see Section D.1 for more details). The remaining parameters are chosen from the literature or to satisfy realistic physical conditions (see Justification column).

We directly estimate the equilibrium contact angle θ_{eq} by measuring the contact angle formed by the shedded group with the floor (see Supplementary Fig. 3D).

The rate of bacterial consumption by the swarm is estimated from the profile of bacterial quantity and swarm height in the travelling phase of the migration (Fig. SM5a) using the travelling wave solution of the model (see Eq. (S19a))

$$B_{ex}(x) \approx B^0 \exp\left[-\frac{E}{U_F}\int_x^0 h_{ex}(\xi)d\xi\right]. \quad \text{[S22]}$$

In writing Eq. (S22), we locate the origin at the position of the peak of the bacterial profile (Fig. SM5a). In Eq. (S22), the constant $B^0 > 0$ accounts for the scaling of the experimental data in terms of bacteria quantity units. We fit Eq. (S22) to the experimental data from Supplementary Fig. 6C using `curve_fit` function in Python, which uses non-linear least square method. Fig. SM5a illustrates a characteristic fit; estimated values of B^0_{exp} and E are given in the figure caption. In Fig. SM5c,

Parameter	Meaning	Value
Ca_κ	Swarm capillary number	≈ 0.174
Ca_ξ	Swarm active capillary number	5.75
R	non-dimension proliferation rate	0.025
L_s	non-dimensional slip length (inverse of the normalised friction with the floor)	0.08
E	non-dimension bacteria consumption rate	0.335
D_c	non-dimensional chemoattractant diffusion coefficient	9.6
Γ_d	non-dimensional chemoattractant decay rate	9.6
H_δ	non-dimensional threshold cell-floor attraction forces	0.0769
$\hat{\alpha}$	Hyperparameter characterising the slope of the alignment function $\hat{S}$	2.
$\hat{m}_B$	Threshold for arrest cell proliferation	0.05
Ca_θ	contact angle capillary number (chosen to match the velocity of the maximum velocity of the swarm in the TW analysis with the result from the dynamical simulations)	0.2

Table SM2. List of the non-dimensional physical parameters in the active thin-film and active droplet models.

we report the estimates of the bacteria consumption rate E for four different sets of experimental (time =50, 55, 60, 65 mins in Supplementary Fig. 6C); for the numerical simulation, we used the average value calculated across these four repeats (see Table SM2).

The slip length ℓ_s and the strength of the cell-floor traction ξ_T are extrapolated from combining the measurement of the bacterial quantity and horizontal cell velocity measured experimentally (last four panels in Fig. S6C). We use Eq. (S7a) to relate the velocity of the cell at the free-surface $\bar{u}_c = u_c(x, h(x,t), t)$ and at the floor $\underline{u}_c = u_c(x, 0, t)$:

$$\bar{u}_c^{ex}(x) = (\underline{u}_c^{ex} - \xi_T s_\alpha^{ex}) \left(1 + \frac{h_{ex}}{2\ell_s}\right) + \xi_T s_\alpha^{ex}. \qquad [S23]$$

Note that while $\bar{u}_c^{ex}$, $\underline{u}_c^{ex}$ and h_{ex} are directly measurable experimentally, we obtain the alignment profile s_α^{ex} as follows. We first use Eq. (S18b) and the fitted bacterial profile to estimate the spatial profile of the chemoattrant, $C^{ex}(x)$. We then use Eq. (S8) to estimate the spatial distribution of the cell alignment s_α^{ex}. Fig. SM5b illustrates the resulting chemoattractant and alignment profiles for the bacterial profile in Fig. SM5a. We then use the `curve_fit` function in Python to fit Eq. (S23) and estimate both ℓ_s and ξ_T. Fig. SM5c illustrates the estimates of the bacteria consumption rate ℓ_s and ξ_T for four different sets of experimental (time =50, 55, 60, 65 mins in Fig. S6C). Additional analysis shows that the estimates for ℓ_s and ξ_T are robust to changes of the hyperparameter $\alpha \in \mathcal{O}(10^{-1}) - \mathcal{O}(10^{1})$. Because it is estimated to be $\approx 10^{-12}$, for the numerical simulation we set $\xi_T = 0$ while we used the average value calculated for ℓ_s across these four repeats (see Table SM2); estimated values are given in Table SM1.

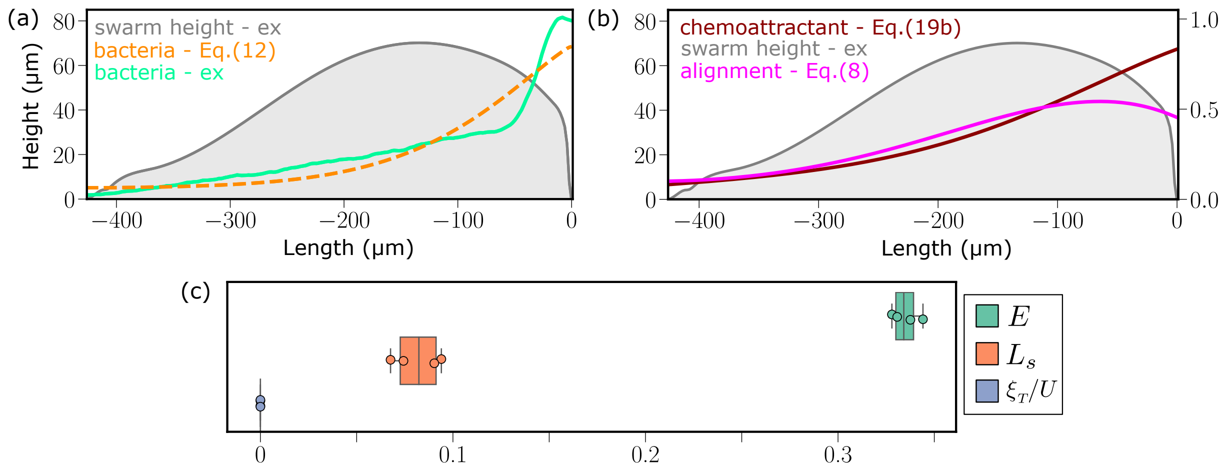

Fig. SM5. (a) Comparison of the experimental data on the bacteria quantity profile and the fitting obtained using Eq. (S22) with parameters estimated via non-linear least-squares: $B^0_{exp} = 68.46\,(a.u.)$, $E = 0.344$. (b) Estimated spatial profile for the chemoattractant C (Eq. (S18b)) and alignment s_α (Eq. (S8)) obtained from the bacteria profile in panel (a) and default parameter values in Table SM2. (c) Estimates for parameter grouping E, L_s and ξ_T/U obtained by fitting the data from Fig. S6C as described in the text. We repeat the fitting considering four different time points (50, 55, 60, 65 mins). Mean values across these four repeats are used as default values in the simulations (see Table SM2).

D.1. Estimating the emergent material properties of the swarm. The emergent material properties of cell groups are characterised by their surface tension (κ), viscosity (η) and activity (ξ). We estimate the values of these parameters matching model predictions with the following metrics characterising the emergent dynamics of the swarm:

1) *time-averaged front velocity*, U_F (Fig. S7B);

2) *distance between shedded groups*, D_g (Fig. S7C).

Looking at Eq. (S7a), the velocity field u_c, and therefore the emergent swarm dynamics, uniquely depend on the ratios of the emergent material properties; specifically, on κ/η and ξ/η. Hence, the three parameters can not be independently identified. Therefore, we focus on inferring their relative size, which is encoded in the two capillary numbers: Ca_κ and Ca_ξ (see Eq. (S12)). To estimate the values of Ca_κ and Ca_ξ from the measurements of the emergent swarm dynamics we proceed as described below.

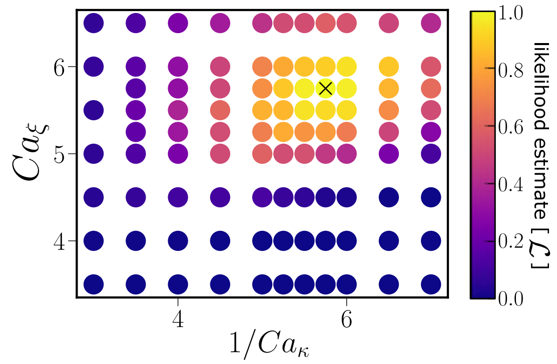

Fig. SM6. Heatmap of the likelihood function $\mathcal{L}$ defined by Eq. (S24); here $\mathcal{L}$ is scaled to take values in the interval $(0, 1]$. Parameters are set to the default values in Table SM2 except for the free parameters Ca_ξ and Ca_κ that we aim to estimate. We highlight in red the parameter values for which no shedding is observed for the whole duration of the simulations. We find that the likelihood is maximised for the set of capillary numbers: $Ca_\xi = 5.75$ and $Ca_\kappa^{-1} = 5.75$, for which $\chi^2 = 0.0032$, $\mathcal{L} \approx 0.996$.

Given a set of parameters (Ca_κ, Ca_ξ), we simulated the thin-film model (see Section B) for an equivalent of 60 hours, in dimensional time units. We estimated the values of U_F and D_g, after discarding the first 30 hours. This is to avoid the results being influenced by the choice of initial conditions. We note that the shedding of the swarm is dependent on the choice of the parameters. Parameter values that yield no shedding are discarded. If shedding is observed in the numerical simulations, we quantified the "goodness-of-fit" to the experimental data by introducing the following likelihood function:

$$\mathcal{L}(Ca_\kappa, Ca_\xi) = e^{-\chi^2(Ca_\kappa, Ca_\xi)}, \quad \text{[S24a]}$$

$$\chi^2(Ca_\kappa, Ca_\xi) = \frac{1}{2}\left(\frac{U_F(Ca_\kappa, Ca_\xi) - \tilde{U}_F}{\sigma_{U_F}}\right)^2 + \frac{1}{2}\left(\frac{D_g(Ca_\kappa, Ca_\xi) - \tilde{D}_g}{\sigma_{D_g}}\right)^2. \quad \text{[S24b]}$$

In Eq. (S24b), the function χ^2 is the standard non-linear least square functional, where $\tilde{U}_F = 4.79\,[\mu\text{m/min}]$ and $\sigma_F = 0.092\,[\mu\text{m/min}]$, and $\tilde{D}_g = 1.25\,[\text{mm}]$ and $\sigma_{D_g} = 0.35\,[\text{mm}]$ are the experimental mean and standard deviation of, respectively, the average front velocity and the distance between the shedded groups. The best fit is chosen as the value of Ca_κ and Ca_ξ that maximises – amongst the tested parameter values – the likelihood function $\mathcal{L}$. Results are shown in Fig. SM6. We are able to identify a unique set of parameters that maximize the likelihood: $Ca_\kappa = 1/5.75$ and $Ca_\xi = 5.75$. This corresponds to the dimensional parameter groupings, κ/η and ξ/η listed in Table SM1.

We can obtain an estimate for the order of magnitude of the activity parameter ξ by relating it to the strength of pulling (or traction) forces that Dictyostelium cells exert on each other, $F_{act} \approx 2 - 10 \times 10^{-8}$ [N/cell] (12). Specifically, we can write $\xi = F_{act} b\rho$, where we can take b to be the characteristic size of pseudopods, which we assume is comparable to the size of a cell $10\,\mu$m, and ρ is directly estimated from the light sheet images (see Fig. 1) $\rho = 9.4 \pm 1.1 \times 10^{-4}$ [cell/μm^3]. Then we find the estimate for the activity $\xi \approx 0.17 - 1.05\,[\text{kPa}]$. This leads to the viscosities in the range $\eta \approx 0.096 - 0.59\,[\text{MPa} \cdot \text{s}]$. Our estimates for the swarm viscosity are in line with estimates reported in (13) for cell monolayers. This can be expected because of the faster dynamics of pseudopod formation/retraction compared to cell-cell adhesion. For the surface tension, we find $\kappa \approx 7.0 - 43.26\,[\text{mN/m}]$, which is within an order of magnitude of the surface tension of water ($\approx 72\,[\text{mN/m}]$).

E. Results.

E.1. Steady simulations: Swarm volume controls mass redistribution during migration. Fig. SM7 illustrates how (a) [(c)] the length and (b) [(d)] the velocity of travelling wave solutions depend on the swarm volume V_{TW}. The different curves in the top and bottom panels correspond to different values of the active capillary number Ca_ξ and the non-dimensional slip length L_s, respectively. We find that for sufficiently high values of the active capillary number Ca_ξ and slip length L_s, a multistability region exists whereby multiple types of travelling-wave solutions exist for the same volume V_{TW}. The region is delimited by two fold points.

This introduces the existence of a critical volume within the system (see dots in Fig. SM7), beyond which only a slow and elongated migration phenotype exists. Below the critical point (see dots in Fig. SM7), capillary forces, which favour a compact swarm, dominate over active forces that favour the elongation of the swarm; hence, travelling-wave solutions are characterised by a compact profile (Fig. 3H main text). Beyond the critical point, active forces dominate over the action of surface tension; hence, travelling-wave solutions are characterised by an elongated highly asymmetric profile (Fig. 3H main text). The transition between these two regimes, which we refer to as elongation transition in the main text, occurs when active and capillary forces balance. When Ca_ξ is small, active forces are too small to compete with the action of surface tension; hence the elongation transition is suppressed. The value of the slip length L_s has also an impact on the bifurcation diagram (Figs. SM7c-SM7d); in particular we find that increasing L_s results in faster moving groups (for equal value of the mass) that delay the transition to the elongated phenotype towards higher volume values. While the location of the critical volume is sensitive to both the value of L_s and Ca_ξ, its existence is much more sensitive to the choice of Ca_ξ than L_s. In the dynamical simulations (Fig. 3B and Movie S6), as the migrating swarm crosses this critical volume it starts elongating and eventually splits, as a result of dewetting. We conclude that the location and existence of the critical mass controls the shedding dynamics. We note that similar patterns have been observed in the study of sliding droplets under gravity (30), where morphological transitions in droplet shape are connected to the phenomenon of pearling (31) – *i.e.*, the emission of smaller droplets from the moving front. While in sliding passive droplets, the driving force – namely gravity, is spatially homogeneous, in our system, the activity term is heterogeneous due to its dependence on the local chemoattractant gradient. This facilitates the elongation and shedding of the droplet by enhancing the asymmetry between the front and rear of the droplet, which in the passive case is only associated with gravity-driven changes in the droplet shape.

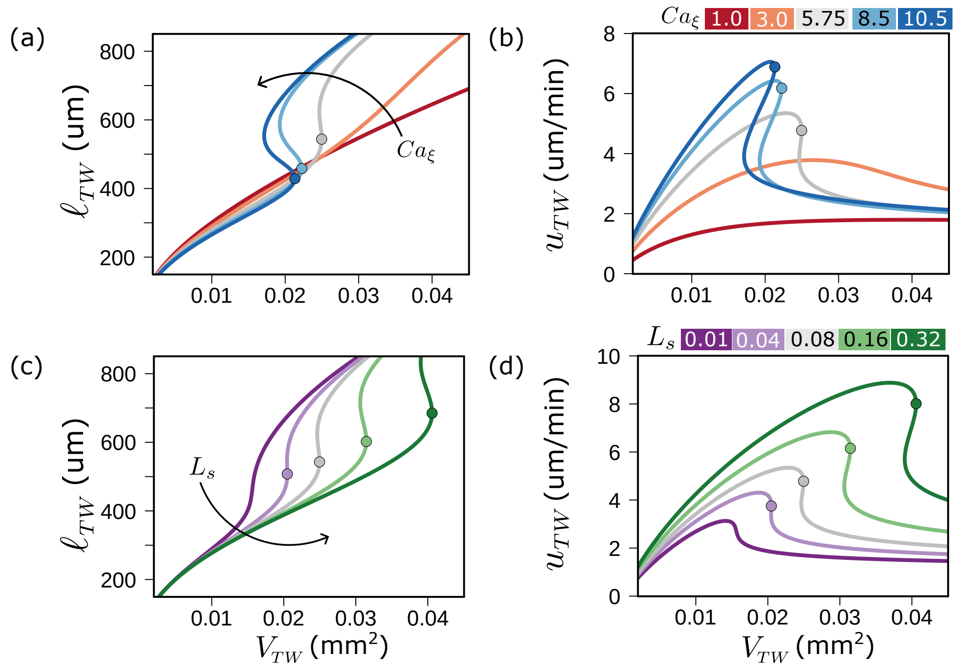

Fig. SM7. The active capillary number Ca_ξ and slip length L_s control the existence of the critical splitting mass. We plot the bifurcation diagrams of travelling-wave solutions of Eq. (S17) in terms of the dependence of the (a) [(c)] length ℓ_{TW} and (b) [(d)] velocity u_{TW} of travelling swarms on the swarm volume, $V_{\mathrm{TW}} = \nu_{\mathrm{TW}} L^2 \tan\theta_e$. Curves of different colour indicates distinct values of (a)-(b) Ca_ξ and (c)-(d) L_s. The dots indicate the location of the right-most fold bifurcation when it exists. Non-dimensional parameters are set to the values in Table SM2. The grey curve corresponds to the bifurcation diagram in Fig. 3G, which was obtained using the fitted values of the model parameters.

E.2. Dynamical simulations: Shedding patterns in growing swarms. The travelling wave analysis (Section E.1) highlights the importance of the swarm material properties in determining whether clump shedding occurs during migration. Dynamical simulations further demonstrate the role of emergent material properties – specifically the passive and active capillary numbers, Ca_κ and Ca_ξ – in the pattern of shedding observed in the dynamical simulations (Fig. 4). In particular, we can identify two types of shedding morphologies: clumps and extended trails. To quantitatively distinguish between these two morphologies, we define the following non-dimensional shape parameters:

$$\mathcal{S} = \frac{\ell}{\sqrt{6V}}. \qquad \text{[S25]}$$

In Eq. (S25), ℓ and V indicate respectively the length and the 2D volume (*i.e.*, area) of the swarm. The scaling used in Eq. (S25) is chosen so that $\mathcal{S} = 1$ for a static droplet at equilibrium. In general, the larger the value of $\mathcal{S}$, the larger the asymmetry in the swarm mass distribution; in particular, an increase in $\mathcal{S}$ correlates with the formation of wide and thin layers of cells at the rear of the leading swarm – characteristic of the extended trail morphology. Generally, $\mathcal{S}$ is a function of time; to generate Fig. 4E in the main text, we use its value just before shedding.

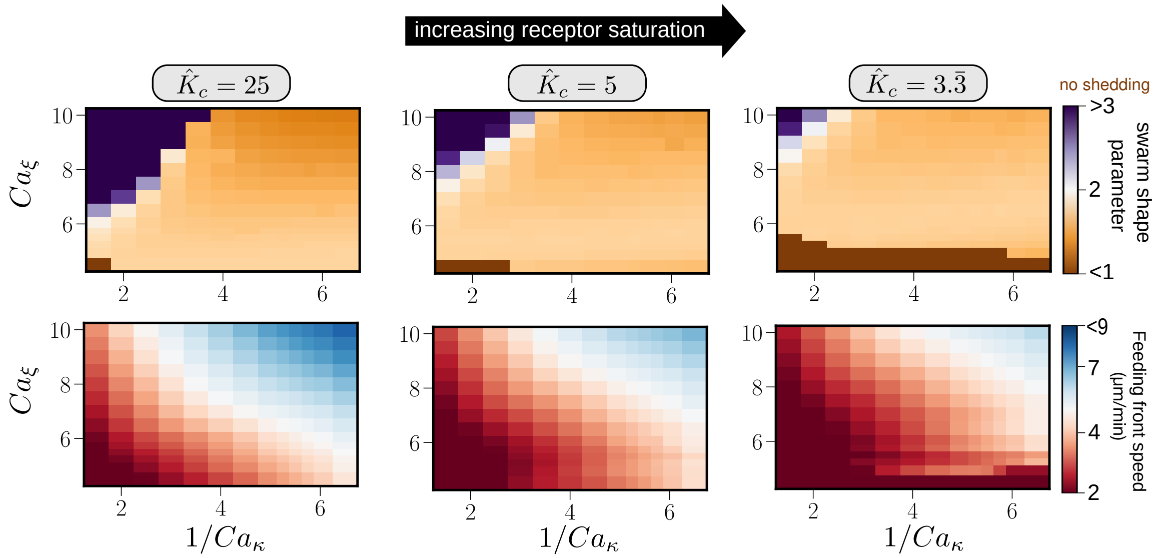

Fig. SM8. Model simulations showing that increasing receptor saturation K_c affects swarm migration and shedding. Panels show phase diagrams for migrating swarms with variable passive and active capillary numbers; Ca_κ and Ca_ξ, respectively. Top: shape parameters for the simulated swarm at the time of shedding; Bottom: average front migration speed for the simulated swarm. Dark brown indicates parameter sets for which no shedding is observed for the duration of the simulation. From left to right, we decrease the value of the non-dimensional dissociation constant $\hat{K}_c = K_c/C_\infty$, to capture the increase in the far-field chemoattractant concentration at higher bacterial densities.

As discussed in the main text, experimental data show an increased propensity for the extended trail morphology and the slowing down of the swarm's migrating front at higher bacterial densities. These observations are explained by greater impedance to cell movement caused by the bacterial lawn at higher bacterial densities, as validated by simulating the model for increasing values of the rescaled slip length L_s. Saturation of the chemoattractant receptors could be an alternative justification for the decrease in the swarm front propagation speed at higher bacterial densities. However, as shown in Fig. SM8, this interpretation contrasts with the observation that increasing bacterial density promotes the extended trail morphology. Following (22), we simulate the effect of receptor saturation on swarm migration by assuming that cell polarisation s_α, defined by Eq. (S8), is a function of gradients in the receptor occupancy,

$$s_\alpha = \tanh\left(\alpha \frac{\partial}{\partial x}\left(\frac{C}{1 + C/K_c}\right)\right). \qquad \text{[S26]}$$

The form of receptor occupancy used in Eq. (S26) can be derived from a single-site equilibrium dissociation equation for the receptor-chemoattractant system, where K_c is the corresponding dissociation constant (32). This introduces a new non-dimensional parameter $\hat{K}_c = K_c/C_\infty$, which measures the ratio between the receptor dissociation constant and the far-field chemoattractant concentration. We investigate how receptor saturation affects swarm migration dynamics by simulating the model for decreasing values of $\hat{K}_c$. As shown in Fig. SM8, receptor saturation can decrease the swarm migration speed, as observed experimentally when increasing bacterial density (Fig. 4C). However, saturation-driven slowing down of swarm migration correlates with reduced shedding and, when shedding occurs is typically characterised by the compact rather than elongated trail morphology. This contrasts with the trend observed when increasing bacterial density in the experiments (Fig. 4B). Consequently, we conclude that receptor saturation alone cannot fully explain the observed swarm dynamics. This prompted us to explore alternative mechanisms. Motivated by the evidence for bacterial impedance to cell motion (see Section "Clump shedding follows gradient dynamics"), we investigated the impact of reducing the slip length on swarm dynamics. We found that this can better explain experimental observations (Fig. 4E).

References

1. P Paschke, et al., Rapid and efficient genetic engineering of both wild type and axenic strains of *Dictyostelium discoideum*. *PLOS ONE* **13**, 1–25 (2018).
2. R Sussman, M Sussman, Cultivation of *Dictyostelium discoideum* in axenic medium. *Biochem. Biophys. Res. Commun.* **29**, 53–55 (1967).
3. DJ Watts, JM Ashworth, Growth of myxamoebae of the cellular slime mould *Dictyostelium discoideum* in axenic culture. *Biochem. J.* **119**, 171–174 (1970).
4. L Eichinger, et al., The genome of the social amoeba *Dictyostelium discoideum*. *Nature* **435**, 43–57 (2005).
5. M Benghezal, et al., Specific host genes required for the killing of *Klebsiella* bacteria by phagocytes. *Cell. Microbiol.* **8**, 139–148 (2006).

6. DM Veltman, G Akar, L Bosgraaf, PJ Van Haastert, A new set of small, extrachromosomal expression vectors for *Dictyostelium discoideum*. *Plasmid* **61**, 110–118 (2009).
7. L Tweedy, et al., Seeing around corners: Cells solve mazes and respond at a distance using attractant breakdown. *Science* **369**, eaay9792 (2020).
8. V Antolović, et al., Transition state dynamics during a stochastic fate choice. *Development* **146**, dev173740 (2019).
9. ER Westbrook, T Lenn, JR Chubb, V Antolović, Collective signalling drives rapid jumping between cell states. *Development* **150**, dev201946 (2023).
10. E Rozbicki, et al., Myosin-II-mediated cell shape changes and cell intercalation contribute to primitive streak formation. *Nat. Cell Biol.* **17**, 397–408 (2015).
11. G Singer, T Araki, C Weijer, Oscillatory cAMP cell-cell signalling persists during multicellular *Dictyostelium* development. *Commun. Biol.* **2**, 139 (2019).
12. E Palsson, HG Othmer, A model for individual and collective cell movement in dictyostelium discoideum. *Proc. Natl. Acad. Sci.* **97**, 10448–10453 (2000).
13. C Blanch-Mercader, et al., Effective viscosity and dynamics of spreading epithelia: a solvable model. *Soft Matter* **13**, 1235–1243 (2017).
14. JS King, RH Insall, Chemotaxis: finding the way forward with dictyostelium. *Trends Cell Biol.* **19**, 523–530 (2009).
15. A Alizadeh Pahlavan, L Cueto-Felgueroso, AE Hosoi, GH McKinley, R Juanes, Thin films in partial wetting: stability, dewetting and coarsening. *J. Fluid Mech.* **845**, 642–681 (2018).
16. A Loisy, J Eggers, TB Liverpool, How many ways a cell can move: the modes of self-propulsion of an active drop. *Soft Matter* **16**, 3106–3124 (2020).
17. S Shankar, V Raju, L Mahadevan, Optimal transport and control of active drops. *Proc. Natl. Acad. Sci.* **119**, e2121985119 (2022).
18. D Moreno-Boza, A Martínez-Calvo, A Sevilla, Stokes theory of thin-film rupture. *Phys. Rev. Fluids* **5**, 014002 (2020).
19. C Pérez-González, et al., Active wetting of epithelial tissues. *Nat. Phys.* **15**, 79–88 (2019).
20. P Pearce, et al., Flow-induced symmetry breaking in growing bacterial biofilms. *Phys. Rev. Lett.* **123**, 258101 (2019).
21. A Dowdell, PI Paschke, PA Thomason, L Tweedy, RH Insall, Competition between chemoattractants causes unexpected complexity and can explain negative chemotaxis. *Curr. Biol.* **33**, 1704–1715 (2023).
22. L Tweedy, DA Knecht, GM Mackay, RH Insall, Self-generated chemoattractant gradients: Attractant depletion extends the range and robustness of chemotaxis. *PLOS Biol.* **14**, 1–22 (2016).
23. EF Keller, LA Segel, Traveling bands of chemotactic bacteria: A theoretical analysis. *J. Theor. Biol.* **30**, 235–248 (year?).
24. M Buenemann, H Levine, WJ Rappel, LM Sander, The role of cell contraction and adhesion in dictyostelium motility. *Biophys. J.* **99**, 50–58 (2010).
25. A Logg, KA Mardal, G Wells, *Automated Solution of Differential Equations by the Finite Element Method: The FEniCS Book*, Lecture Notes in Computational Science and Engineering. Vol. 84, (2012).
26. D Peschka, Variational approach to dynamic contact angles for thin films. *Phys. Fluids* **30**, 082115 (2018).
27. R Veltz, BifurcationKit.jl (2020) https://github.com/rveltz/BifurcationKit.jl.
28. P Gaudet, P Fey, R Chisholm, Growth and maintenance of dictyostelium cells. *Cold Spring Harb. Protoc.* **12**, pdb.prot5099 (2008).
29. A Kashyap, W Wang, BA Camley, Trade-offs in concentration sensing in dynamic environments. *Biophys. J.* **123**, 1184–1194 (2024).
30. S Engelnkemper, M Wilczek, SV Gurevich, U Thiele, Morphological transitions of sliding drops: Dynamics and bifurcations. *Phys. Rev. Fluids* **1**, 073901 (2016).
31. T Podgorski, JM Flesselles, L Limat, Corners, cusps, and pearls in running drops. *Phys. Rev. Lett.* **87**, 036102 (2001).
32. EA Ferguson, J Matthiopoulos, RH Insall, D Husmeier, Inference of the drivers of collective movement in two cell types: Dictyostelium and melanoma. *J. The Royal Soc. Interface* **13**, 20160695 (2016).
